# Combined Modeling of Multiple Exposure Routes for Terrestrial Arthropods Using the Toxicokinetic–Toxicodynamic BufferGUTS Model

**DOI:** 10.1021/acs.est.5c03925

**Published:** 2025-07-29

**Authors:** Leonhard U. Bürger, Florian Schunck, Andreas Focks

**Affiliations:** 9186 Osnabrück University, Barbarastr. 12, 49076 Osnabrück, Germany

**Keywords:** TKTD, toxicokinetic–toxicodynamic, GUTS, environmental risk assessment, terrestrial arthropod, nontarget arthropod, NTA, mechanistic effect modeling

## Abstract

Pesticide applications in agricultural landscapes pose significant risks to nontarget arthropods (NTAs), which are vital for maintaining ecological functions such as pollination and pest control. Effective risk assessment is made challenging by the complexity of terrestrial exposure, which is highly variable and event-driven. Unlike in aquatic settings, NTAs experience pesticide exposure through multiple routes, such as overspray, ingestion, or contact. To integrate such multiple exposure routes into a unified framework, this study proposes an extension to the toxicokinetic–toxicodynamic (TKTD) and time-resolved BufferGUTS model that can be applied to a broad range of NTAs without species-specific adaptations. Our model extension draws on principles from mixture toxicity and allows for individual effect contributions and effect kinetics per uptake route, providing further insight into the substance effect dynamics. We tested the model using honeybee () data for topical and oral exposures to five insecticides. Among the insecticides tested, only one exhibited different kinetics between topical and oral applications, while three exhibited nonsimilar effects from the two routes. We recommend the integration of such TKTD models into environmental risk assessment practices to combine uptake route effects, model field-relevant exposure scenarios, and identify the most relevant routes for each substance. This will enable more informed decision-making, thereby enhancing ecological management and protection efforts.

## Graphical abstract

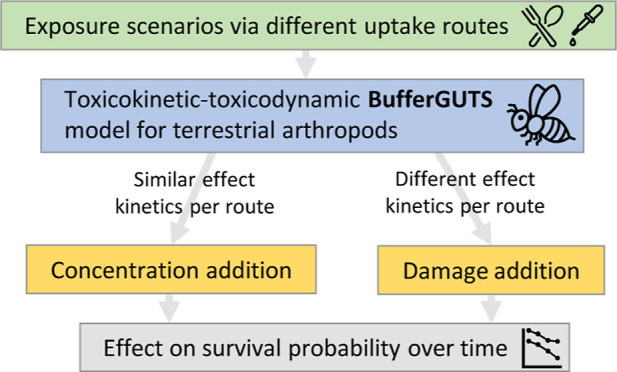

## Introduction

Pesticide applications in agricultural landscapes can harm nontarget species unintentionally, with nontarget arthropods (NTAs) being particularly vulnerable.^1–3^ Protecting NTAs is imperative for maintaining key ecological functions, such as pollination and pest control,^4^ and for preserving biodiversity within agricultural ecosystems.^5^ However, including above-ground NTAs in environmental risk assessment poses significant challenges due to the complexities of quantifying exposure in terrestrial environments. Unlike in aquatic settings, where the exposure to pesticides is mostly uniform and can be measured as a concentration in water, terrestrial exposure is highly variable and event-driven. NTAs can encounter pesticides through a variety of pathways, such as direct overspray during application, ingestion of contaminated material, or contact with treated surfaces. The lethality of these exposures can vary depending on the route, with each route potentially having a distinct lethal concentration.^6^ Furthermore, exposure events can occur in isolation, concurrently, or in succession, adding additional layers of complexity to predictions of effects and, therefore, to an accurate risk assessment.

To address nonconstant exposure scenarios and time-resolved effects, toxicokinetic–toxicodynamic (TKTD) models have been developed, like the General Unified Threshold model of Survival (GUTS).^7,8^ These models establish mechanistic links between substance exposure and survival by simulating the uptake and time-resolved effects of substances. In aquatic environments, numerous TKTD case studies have been conducted,^7,9–11^ and GUTS models are considered ready for application in aquatic environmental risk assessment.^12^ In the context of above-ground terrestrial arthropods, two models have addressed the issue of nonconstant and event-driven exposures: the BeeGUTS model,^13,14^ designed specifically for bee species by incorporating species-specific information to preprocess exposure, and the species-independent BufferGUTS model.^6^ The additional combination of effects from different uptake routes has, to date, been realized only in the BeeGUTS model, which assumes equal effect contributions from all pathways. While combined effects from multiple uptake routes are not yet part of standard risk assessment procedures, TKTD models provide the potential to account for them so that they could be considered in the future to more accurately reflect actual field exposures. Current lower-tier risk assessment procedures typically only consist of laboratory tests for individual routes in isolation and then focus on the most conservative results.^15,16^ Focusing risk assessment on single uptake routes disregards the potential combined effects of a substance concentration taken up via multiple uptake routes simultaneously, as is the case in actual field situations. This could lead to application concentrations that are conservative in single uptake route experiments no longer being conservative when individuals are exposed to the same concentrations via multiple uptake routes.

To help address this issue, we propose an extension to the BufferGUTS model that integrates multiple exposure routes within a unified framework. It can be used with typical existing (laboratory) data sets for terrestrial arthropods and was tested using data for the honeybee . Our approach draws inspiration from established principles of mixture toxicity for substances with a similar mode of action^17–19^ and allows for dissimilar effect contributions from the different routes. By extending the model in this manner, we aim to enhance the accuracy of risk predictions for arthropods under real-world conditions and deepen our understanding of how specific uptake routes contribute to the overall impact of insecticide exposure in terrestrial contexts. Importantly, the model is designed to be universally applicable to a wide range of NTAs without requiring species-specific adaptations. This methodology is expected to yield valuable insight into how pesticide substance effects behave depending on the route via which individuals are exposed. This insight can aid in the development of more robust risk assessment frameworks and foster strategies that minimize adverse effects on NTA while ensuring effective pest management practices.

## Materials and Methods

### General Model

The General Unified Threshold model of Survival (GUTS)^7,8^ establishes a mechanistic link between exposure and survival through toxicokinetic-toxicodynamic (TKTD) modeling. In scenarios where internal concentrations are unavailable, reduced GUTS models provide an alternative. These simplified models use a dominant rate constant *k*_d_ to describe the uptake and elimination of chemicals, connecting external concentrations *C* to a state variable known as scaled damage *D*. This damage is coupled to one of two death mechanisms: stochastic death (SD) or individual tolerance (IT). Expanding on this concept, the BufferGUTS model^6^ introduced an additional buffer state *B* to better capture the dynamics of above-ground terrestrial exposures, which may reflect residues on the exoskeleton or within the stomach of an organism.

In this study, we extend the BufferGUTS model to accommodate multiple uptake routes. Our approach draws on the classical theory of mixture effect^17,18^ and GUTS mixture modeling approaches.^19^ Unlike traditional mixture assessments, which involve multiple substances, our focus on a single chemical allows us to assume a similar mode of action across different uptake routes. This diverges from the “independent (joint) action” models,^18,19^ which are therefore not regarded. Classic mixture models for similar modes of action predict cumulative effects by summing (scaled) exposure concentrations to predict the effect of the mixture. However, the time-resolved nature of TKTD models allows differentiation between different types of effect kinetics. Some exposures may result in fast kinetics, with effects on mortality becoming visible already shortly after exposure, while others display slower kinetics with delayed effects. These differences can depend on both the uptake route and the specific characteristics of the substance in question.^6^ For substances with the same time period between exposure events and mortality for all uptake routes, similar kinetics can be assumed, and one dominant rate constant *k*_d_ can be used to describe uptake and effect over time. When all routes share the same kinetic profile, it allows for the weighted summation of exposure concentrations *C*_1_···_*N*_ or buffer concentrations *B*_1_···_*N*_ before the damage *D* is calculated. Since the parameters (buffer speed constant η and dominant rate constant *k*_*d*_) that govern the transfer from exposure concentrations *C*_1_···_*N*_ to buffer concentrations *B*_1_···_*N*_ are consistent across all uptake routes, it does not matter which of the two is used for the summation. Such models using the same dominant rate constant *k*_d_ for all uptake routes are hereafter called concentration addition (CA) models (see Figure 1, left).

**Figure 1:**
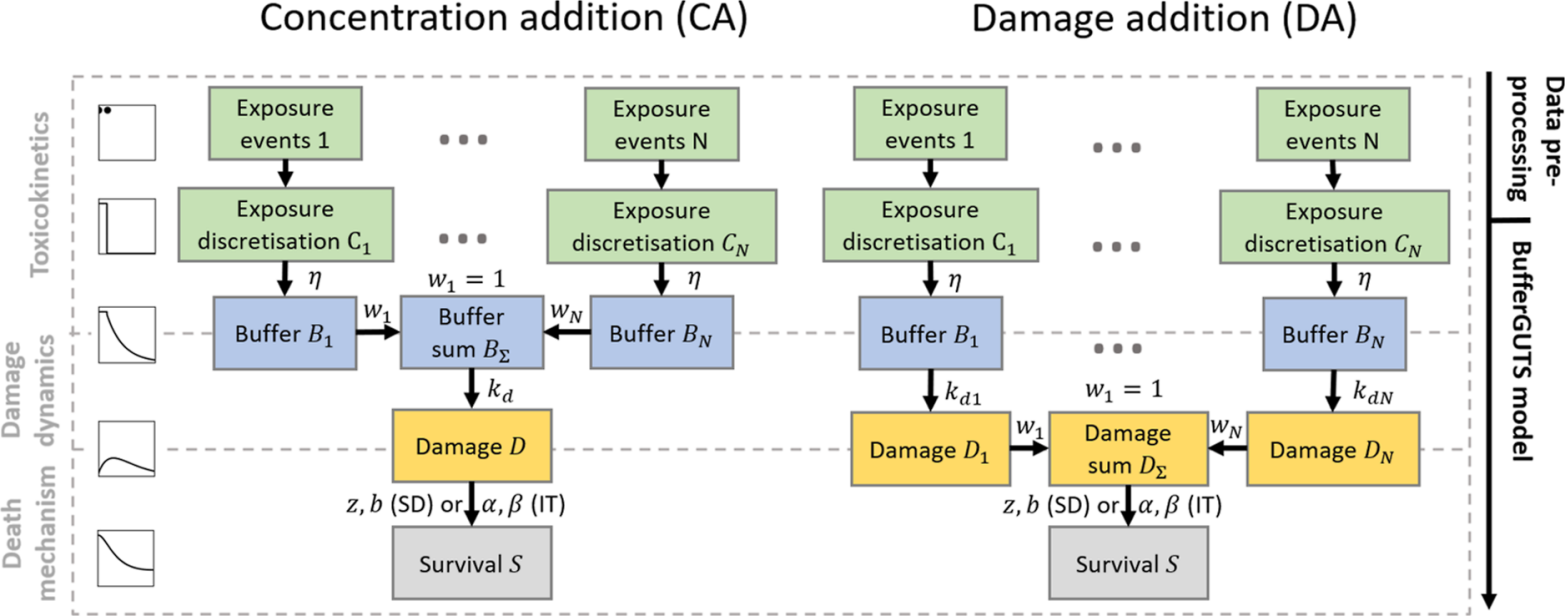
Schematic overview of the BufferGUTS variants combining multiple exposure routes. Both variants can be used with either the stochastic death (SD) or individual tolerance (IT) death mechanism. Shown weights *w*_1···*N*_ can be applied to convert exposure units into one unit at any state before they are combined.

When different uptake routes result in distinct kinetics of observable effects, individual dominant rate constants *k*_d1···*N*_ are required for each route. This scenario occurs, for example, when a substance exhibits faster effects upon ingestion compared with topical application. Subsequently, the different exposure routes are combined through a weighted summation of the damages *D*_1···*N*_ for each route. These models are therefore referred to as damage addition (DA) models (see Figure 1, right).^19^

The weights *w*_1···*N*_ used for aggregating concentrations (CA) or damages (DA) account for exposure-route-specific units and sensitivities. By setting *w*_1_ = 1, we ensure parameter identifiability and align the units of the state variables (buffer sum *B*_∑_ and damage *D*) and parameters (threshold *z* and median of distribution α) to the exposure unit of uptake route 1. Our DA implementation follows the mixture modeling framework outlined by Bart et al.^19^ with the addition of the buffer states *B*_1_···_*N*_. The reduced GUTS and BufferGUTS models have 3­(+1) parameters, which means three parameters govern the TKTD dynamic and a background mortality *h*_b_. Our CA models have one additional weight parameter *w*_2···*N*_ per uptake route beyond the first, resulting in *N* + 2­(+1) parameters for *N* uptake routes. DA models need additional dominant rate constants *k*_*d*2···*N*_ and weights *w*_2···*N*_ per additional uptake route, resulting in 2*N* + 1­(+1) parameters for *N* uptake routes. For only one uptake route (*N* = 1), both the CA and DA models are equal to the BufferGUTS models with 3­(+1) parameters. For two uptake routes (*N* = 2), CA models have 4­(+1) and DA models have 5­(+1) parameters. Depending on the species and experiments available, the number of relevant uptake routes can vary. For most above-ground arthropods, at least two uptake routes are of importance: contact and oral. Oral exposures include all compounds taken up via feeding and can include “acute”^20^ or “chronic”^21^ feeding experiments, meaning exposure over short or long periods of time. Sources of contact exposure can vary substantially, depending on the species and test experiments used. They can include topical applications on individuals via droplets^22^ or spraying devices and sustained contact with exposed surfaces or environments, such as glass plates,^15^ soil, or (nondigestible) plant material.

### Bee Data Sets

The data set used for this study was derived from standard regulatory reports for the honeybee available through the Bayer transparency initiative (for report numbers, see Table 1). The data set used is a subset of the reports used in the BeeGUTS^13^ and BufferGUTS^6^ analyses. For our analyses, we selected those substances for which reports on acute oral, acute contact, and chronic oral tests are available, with a mortality of at least 30% in one of the replicates. The resulting data set includes five substances: deltamethrin (pyrethroid, 5 tests), ethiprole (phenylpyrazole, 5 tests), imidacloprid (neonicotinoid, 9 tests), tetraniliprole (anthranilic diamide, 5 tests), and thiacloprid (neonicotinoid, 5 tests), shown in Table 1. In acute oral tests,^20^ food solutions with varying concentrations of compounds were fed for up to 6 h, and uncontaminated food was provided later. Chronic oral tests^21^ lasted for 10 days, with daily replenishment of contaminated food solutions. For acute contact tests,^22^ dissolved compound doses were applied directly to the bee thorax. All tests were performed with batches of ten bees, and their survival was recorded at least daily for two (acute) to 10 days (chronic). The survival numbers of all repetitions of a given treatment were summed to reduce the number of evaluations of the likelihood function without affecting the result.^8^ The exposure events described for all tests were discretized based on Bürger and Focks (2025),^6^ with the smallest time unit for exposures being 1 h. This smallest time unit means that all exposures shorter than an hour are still modeled as lasting for an hour. This was implemented in the original model to assign instant exposures, such as overspray, to an area under the curve for modeling. Exposure is therefore considered constant for 1 h and zero thereafter in acute contact data sets, constant during the feeding period of up to 6 h and zero afterward for acute oral data sets, and constant throughout the test period for chronic data sets. Three acute oral tests were excluded from the final data set because their LD50 values after 48 h differed by factors of eight or more from the other acute oral tests and were considerably lower than the chronic oral LD50 after 10 days for the same substances (see Table 1). In total, 26 tests from 19 different reports comprise the database for this study.

**Table 1:** List of Used Standard Regulatory Tests Including Acute Contact (AC), Acute Oral (AO), and Chronic Oral (CO) Exposures and Their LD50/LC50 Values for the Honeybee ^a^

compound	report no.	exposure	48 h LD50	10 day LD50	48 h/10 day LC50
			(μg a.i./bee)	(μg a.i./bee/day)	(mg a.i/kg food)
Deltamethrin	M-149494	AC	0.262		
Deltamethrin	M-444971	AC	0.119		
Deltamethrin	M-149496	AO	1.45		72.4
Deltamethrin	M-444971	AO*	0.164*		8.21*
Deltamethrin	M-477250	CO		0.648	18.0
Ethiprole	M-192387	AC	0.0127		
Ethiprole	M-214951	AC	0.0540		
Ethiprole	M-192387	AO	0.0336		1.68
Ethiprole	M-214951	AO	0.0327		1.64
Ethiprole	M-581904	CO		0.000695	0.0478
Imidacloprid	M-006940	AC	0.0819		
Imidacloprid	M-067751	AC	0.0445		
Imidacloprid	M-068023	AC	0.0728		
Imidacloprid	M-006940	AO*	0.00348*		0.174*
Imidacloprid	M-016942	AO	0.0763		3.81
Imidacloprid	M-067751	AO	0.0945		4.73
Imidacloprid	M-067996	AO	0.136		6.80
Imidacloprid	M-068023	AO	0.102		5.08
Imidacloprid	M-600686	CO		0.128	1.32
Tetraniliprole	M-438810	AC	0.993		
Tetraniliprole	M-441758	AC	1.34		
Tetraniliprole	M-438810	AO	0.106		5.28
Tetraniliprole	M-441758	AO*	0.0107*		0.537*
Tetraniliprole	M-551955	CO		0.0173	0.710
Thiacloprid	M-000856	AC	40.7		
Thiacloprid	M-001004	AC	44.2		
Thiacloprid	M-000856	AO	17.5		873
Thiacloprid	M-001004	AO	13.7		684
Thiacloprid	M-475374	CO		2.99	48.9

a Listed LD50 and chronic oral 10 day LC50 values were calculated from extracted report values. All 48 h LC50 values for acute oral tests were estimated by assuming a consumption of 20 mg/bee during the exposure period. The three tests marked with * were excluded from the analysis because their LD50 value differs from the other tests for the same compound and exposure. The full study reports can be requested by sending an email with the report numbers to cropscience-transparency@bayer.com.

### Exposure Unit

Reduced GUTS models can work with all types of exposure units because all state variables and parameters are expressed in units related to the exposure unit. When different test designs are combined in one model, they all need to share the same exposure unit, or additional weight parameters are required to convert the exposure values and units. Both acute tests are reported in doses of active ingredient (a.i.) per bee (μg a.i./bee); chronic oral reports include doses per bee per day (μg a.i./bee/day) and the concentration of the substance in the provided food (mg a.i./kg food). Intuitively, converting all exposures to doses seems to be the simplest option, but doses in chronic tests have different consequences compared to acute tests. In acute tests, the whole dose is consumed or applied to the organism in a short amount of time before substantial effects or degradation can occur. In chronic oral tests, lasting for 10 days, reported doses are the sum of the daily consumption of the compound, averaged over the exposure period. Thus, such tests include effects like food avoidance for higher compound concentrations (e.g., ethiprole with a food consumption of 41.0 mg/bee/day in the control and 13.8 mg/bee/day at the highest tested concentration) and do not account, for example, for degradation or excretion. Due to these differences, acute and chronic oral doses cannot be seen as equal, and units cannot be easily converted into each other. To solve this problem, we suggest using the unit of the exposed food solution used in oral tests as the unit for oral exposures instead of the consumed compound dose. This unit is independent of exposure time and is directly correlated with the concentration of the compound in the stomach, which can be assumed to be the relevant exposure to the individual.^13^ For chronic oral tests, the corresponding values are reported, and for acute oral tests, they can be calculated from the dose in combination with the amount of food consumed during the exposure phase. Since consumed food amounts are not always included in the acute oral laboratory reports used, the same consumption was assumed for all tests. The reported consumption numbers were relatively constant, around 20 mg of food per bee, while more recent reports state additionally that the experiments were aimed at a consumption of 20 mg of food per bee. Therefore, we used the value of 20 mg of food per bee for all tests, and compound concentrations in food solutions for acute oral tests were accordingly calculated by dividing the dose reported by 20 mg of food per bee (see LD50/LC50 values in Table 1). Exposure in acute contact tests is not converted and used in the reported form as a dose per bee.

The used data set thus covers the two uptake routes: contact and oral. Oral exposures are associated with acute and chronic feeding experiments in the unit mg a.i./kg food, and contact exposures are assigned to acute topical application in the unit μg a.i./bee. In addition, contact exposure was chosen to be exposure no. 1, thus making μg a.i./bee the unit of states and parameters not related to oral exposure. Since only two uptake routes are considered, we use a simpler subscript for some parameters hereafter. Weight *w* is the weight to convert oral exposure units into contact units, and the dominant rate constants in DA models are *k*_d_ for contact and *k*_d,oral_ for oral exposures.

### Choice of Model Variants

For various species and compound combinations, one model variant may be more suitable than another. To determine which model variant is preferred for each combination, we initially calibrated the CA and DA models with their IT and SD death mechanisms to all data. Next, we used different model evaluation criteria to assess their suitability. On the one hand, we used the Bayesian information criterion (BIC),^23^ and on the other hand, we used the normalized root-mean-square error (NRMSE). The BIC compares the likelihood L of a model to the number of free parameters *k* and data points *n* as BIC=k·ln(n)−2log(L) for a given data set, with lower values indicating a preferable model. Thus, it considers the trade-off between model accuracy and the number of parameters between the CA and DA model variants. It should be noted that BIC values can only be used to compare model performance within one data set because likelihood L values of calibrations are specific to data sets and are not normalized. Because both SD and IT death mechanisms use the same number of parameters, we also use NRMSE to compare the accuracy directly. It is calculated by normalizing the root of the mean square error between predicted (*y*_*i*_) and observed individual numbers ${ŷ}_{i}$ with the mean number of observed individuals y̅ as $NRMSE=1/\mathrm{y̅}\cdot \sqrt{1/n\cdot \sum_{i=1}^{n}{\left(y_{i}-{ŷ}_{i}\right)}^{2}}$ . It normalizes the model error over all observations 1···*n*, enabling a comparison between models and data sets with values close to zero indicating a good fit. If multiple models perform comparably well for the two criteria, the parameter uncertainty index (PUI)^24^ is additionally considered. PUI is a metric of the average parameter uncertainty in the calibrated model across all nonbackground mortality parameters. It is calculated as $PUI=\frac{1}{k-1}\sum_{i=1}^{k-1}\log_{10}\left(Q97.5_{i}/Q2.5_{i}\right)$ from the log_10_ of the ratio between the 97.5th and 2.5th quantile *Q* for each free considered parameter *i*. The sum of all of the ratios is then divided by the number of considered parameters *k* – 1 (the background mortality *h*_b_ is not considered in the PUI). High PUI values for calibrations with low NRMSE uncertainties can indicate calibrations with parameter correlations because different parameter combinations can result in comparable fits. Therefore, values close to zero are ideal for this index due to its implicit indication of low parameter correlations and other undesirable characteristics.^24^

### Model Implementation and Calibration

All four model variants were implemented utilizing functions of the pymob (github.com/flo-schu/pymob) package, which uses *numpyro* ^25^ with the neural ODE solver *diffrax* ^26^ to provide the gradients for parameter inference in ODE systems. Within the framework, Bayesian parameter estimation was performed using the Markov chain Monte Carlo (MCMC) sampler NUTS.^27^ We used preliminary calibrations using stochastic variational inference (SVI)^28^ to determine priors for the parameters of each model. The final MCMC inference process was set to use 16 chains with 2000 warm-up and 2000 draw steps. Some Markov chains got stuck on pseudolocal minima during warm-up with very small step sizes of the sampler and very low variance in the sampled parameters and considerably worse likelihoods. We currently assume that this is due to problematic interactions between numeric errors below the tolerance thresholds of the ODE solver and the behavior of the MCMC algorithms to explore variations in the likelihood space. Since the chains do not seem to contain relevant regions in the likelihood space, we chose to exclude these chains. Thus, only chains with minimum parameter standard deviations above 10^–6^ and a mean likelihood less than 100% worse than the mean likelihood of the best chains were selected for further analysis.

## Results and Discussion

### Comparison of Model Variants

All four BufferGUTS model variants were successfully calibrated to all substance data sets. The three model performance metrics used for the analysis are shown in Figure 2, where NRMSE and BIC follow the same trends and are consistent concerning the best model(s) across all data sets. The PUI also shows comparable trends but differs for some model–substance combinations from the ratings of the other two metrics. The values of the individual calibrated parameters and uncertainties are visualized in the Figures S1–S5 and given in Table S2.

**Figure 2:**
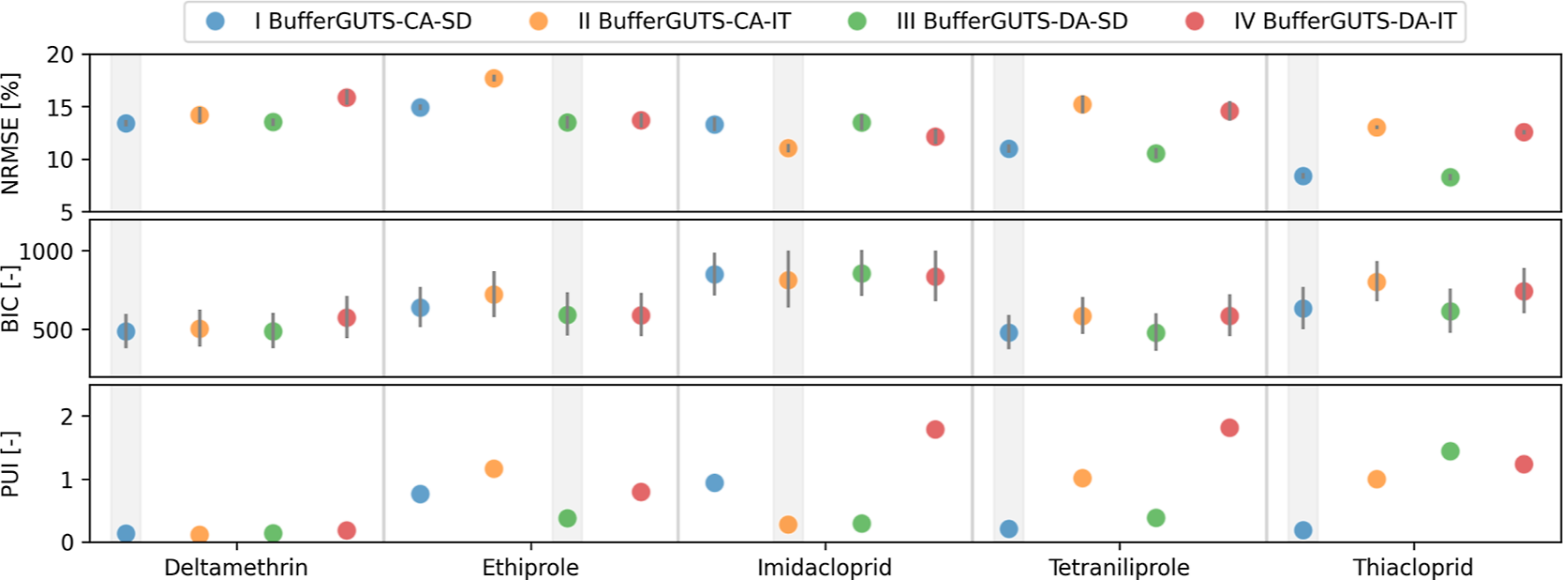
Visualization of the normalized root-mean-square error (NRMSE), the Bayesian information criterion (BIC), and the parameter uncertainty index (PUI) for BufferGUTS models with concentration addition (CA) and damage addition (DA) and the two death mechanisms—stochastic death (SD) and individual tolerance (IT). Shown uncertainties represent the 95% credibility interval, lower metric values are favorable, and the best-performing models for each substance are underlaid in gray.

For the deltamethrin data set, all models perform almost equally well across all metrics; thus, all models could be used to model the substance. BufferGUTS-CA-SD performs best in the NRMSE and BIC metrics and shows a very good PUI (0.13), so that it is selected as the overall best model for the substance. The other four substances show favorable metric values for models with certain combinations of uptake routes and death mechanisms. For ethiprole, models with damage addition (DA) outperform the concentration addition (CA) models, indicating different kinetics for contact and oral exposure. Since the PUI for the DA model with a stochastic death (SD) death mechanism is considerably lower than its individual tolerance (IT) counterpart, BufferGUTS-DA-SD is chosen as the best model for ethiprole. Performance metrics for imidacloprid favor models with the IT death mechanism, and consideration of the PUI indicates BufferGUTS-CA-IT to be the best calibration. Models with the SD death mechanism outperform the IT models for tetraniliprole and thiacloprid in performance metrics. For thiacloprid, BufferGUTS-CA-SD shows a considerably better PUI and is thus selected for the substance. The case for tetraniliprole is more complicated because the DA model has a better mean NRMSE (CA: 10.96%, DA: 10.52%) and the CA model has a better PUI (CA: 0.21, DA: 0.38). We favor the model with fewer parameters in this case, thus also selecting BufferGUTS-CA-SD for this substance.

Exemplary calibration result subsets for deltamethrin and tetraniliprole are shown in Figure 3. All four models were able to capture the time course of the survival *S* data points for both substances. Looking further at the time courses of buffer *B* and damage *D* reveals differences in the models and the effects of the substance. As mortality occurs for deltamethrin already in the first 24 h, all dominant rate constants are relatively quick, and the buffer *B* and damage *D* are (close to) zero after a couple of days. For tetraniliprole, with mortality starting only after two to 3 days, dominant rate constants are lower; thus, the buffer *B* and damage *D* states need considerably longer to get back to zero. Different effect speeds also influence the spread of predictions for the time after survival data points were collected in acute experiments. With fast effect kinetics and thus also quick damage *D* depletion, all models equally predict no further mortality after a couple of days. For slower kinetics, damage *D* values are still high or even increase when the observation period ends, so survival predictions beyond the observation period differ between models. Due to their death mechanism, related to the maximum damage *D*, IT models have lower dominant rate constant values and therefore a delay in damage *D* accrual and depletion as compared to SD models (see Figures S1–S5). The mentioned trends related to effect kinetics are thus mainly observable when models are compared to other models with the same death mechanism. Comparable observations can also be made for the other substances and concentrations (Figures S6–S11).

**Figure 3:**
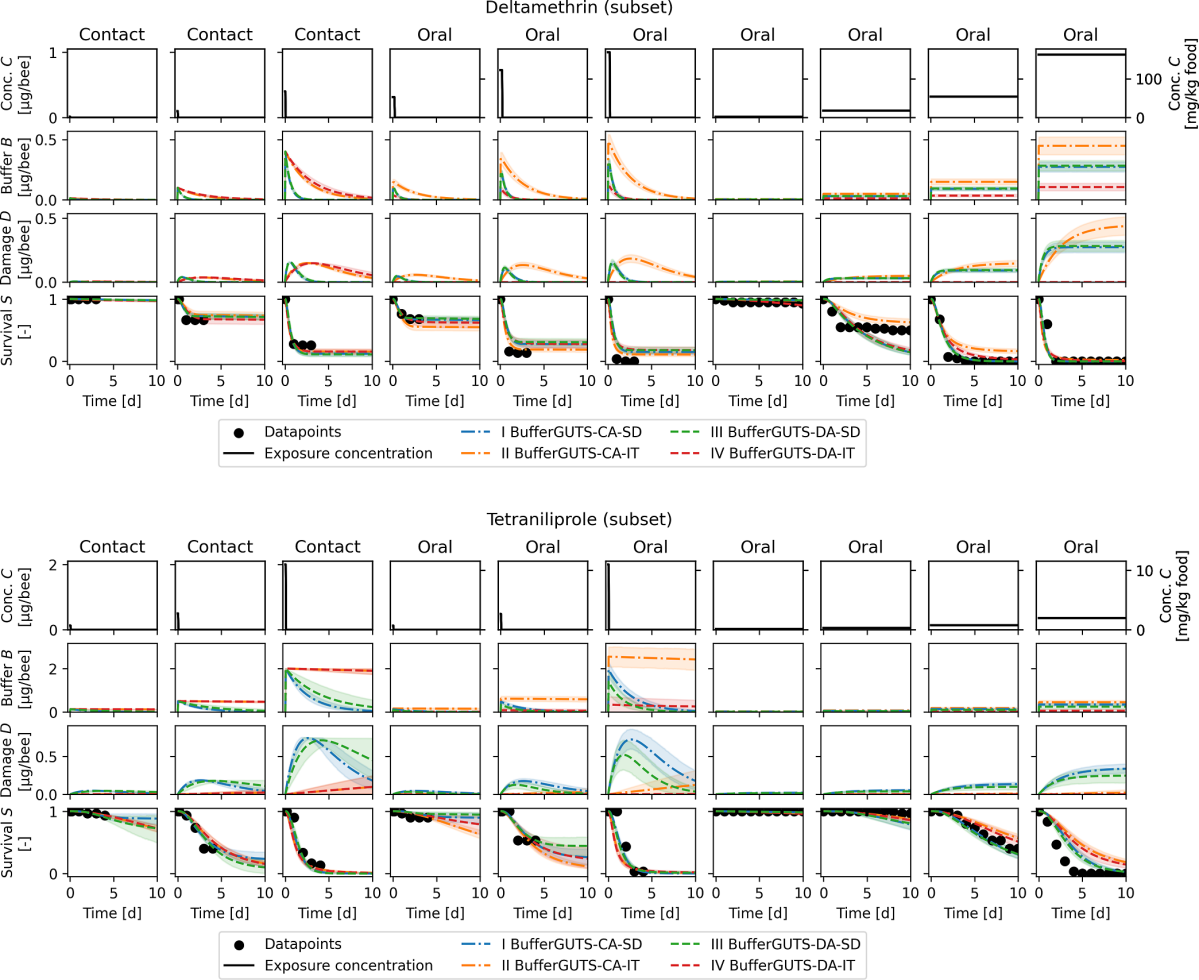
Subset of the calibration results for deltamethrin (top) and tetraniliprole (bottom) for the four BufferGUTS variants with concentration addition (CA) or damage addition (DA) exposure route combination and stochastic death (SD) or individual tolerance (IT) death mechanisms. Uncertainties shown are the 95% credibility intervals, and calibration results for all concentrations are shown in the Figures S6−S10. Exposure route weights *w* are already applied when concentrations *C* are converted into the buffer *B* in this figure to ease readability.

### Contributions of Uptake Routes to the Effect

The susceptibility to exposure via oral or contact exposure varies among the different substances. We analyzed this based on the calibrated weights *w* that are used in the model to convert oral exposure units into contact exposure units (Figure 4). If these calibrated weights *w* are equal to the conversion factor of 20 mg of food per bee that was used to preprocess acute oral doses, then the amounts of substances have the same effect, regardless of whether they were applied to the thorax or were ingested. This can be seen for ethiprole and for the best-fitting model for imidacloprid. In contrast, for substances like deltamethrin, the weights *w* were considerably lower than the conversion factor, meaning that the ingested amounts of the substance had a lower effect on individuals than the applied doses. If weights *w* are higher than the conversion factor, the results indicate that doses that are taken up orally have a higher effect than the same doses applied via contact exposure, as is visible for tetraniliprole and thiacloprid. The variants of the model with concentration addition (CA) have no additional uptake route-specific parameters; therefore, all the differences in the contribution of the uptake route effect contribution are bundled in the weight *w* parameter. Models with damage addition (DA) also differentiate between dominant rate constant *k*_d_ for contact exposure and *k*_d,oral_ for oral exposure. Therefore, additional differences in susceptibility to the uptake route are found in the two rate parameters. Lower dominant rate constants *k*_d_ or *k*_d,oral_ lead to lower maximum damage *D* because less of the concentration in the buffer *B* is already converted into damage when the exposure concentration *C* goes back to zero in acute experiments. Interestingly, the difference between the contact and oral dominant rate constants is larger for DA models with the individual tolerance (IT) death mechanism than for DA models with the stochastic death (SD) death mechanism (see Figures S1–S5). The death mechanisms themselves only relate to the damage and are thus independent of the uptake route.

**Figure 4:**
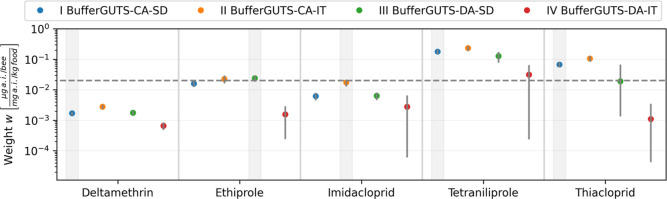
Calibrated weight *w* parameter values for all substances for the four BufferGUTS variants with concentration addition (CA) or damage addition (DA) exposure route combination and stochastic death (SD) or individual tolerance (IT) death mechanisms. The weight *w* is the calibrated conversion factor from oral exposure in mg a.i./kg food to exposure in μg a.i./bee as used for contact exposures. Shown parameter uncertainties are the 95% credibility intervals, and the best-performing models for each substance are underlaid in gray. The dashed horizontal line corresponds to the 20 mg food/bee used to convert acute oral doses to food concentrations in the data preprocessing.

The impact of uptake routes on the effect strength can already be roughly estimated by looking at acute LD50 values (Table 1). However, the analyses based on BufferGUTS use all available acute and chronic data sets and thus provide more robust and accurate results. They can identify uptake routes with the highest susceptibility as well as ratios between susceptibilities based on the calibrated model parameters and in that way provide a deeper understanding of the processes and, eventually, of the importance of the single uptake routes across chemicals.

### Conversion of Acute Oral Units

The comparison between contact and oral effects is based on the assumed conversion factor of 20 mg of food consumed per bee in acute oral tests. If such a conversion factor cannot be defined to unify acute and chronic oral exposures, then each can be individually modeled as a distinct uptake route. This could be the case if the substance already shows severe effects or avoidance behavior during the acute feeding period. Modeling such exposures as individual routes allows for combined effect predictions and the analysis of individual uptake route effect contributions.

However, it is important to note that differentiating between acute and chronic oral exposures by modeling them as separate uptake routes may not be biologically valid. This differentiation should only be done if biologically comparable exposures cannot be converted to a similar unit. Artificially separating these exposures complicates predictions for nonstandard exposure periods, such as a 2 day exposure, because these distinct routes are calibrated specifically for acute (up to 6 h) or chronic (10 days) exposure durations and rely on their respective units. Such an artificial separation thus limits the flexibility and accuracy of effect predictions in nonstandard exposure scenarios and should be avoided.

## Prediction of Unseen Data

### Prediction of Acute Effects from Chronic Data

A true validation of our model results with experimental observations of mortality over time resulting from multiple exposure peaks and a combination of exposure via different uptake routes was not performed due to a lack of such data. Instead, we used the acute oral data sets to test parts of the model structure by calibrating the best-performing model variants for each substance to just the acute contact and chronic oral data. The resulting models are then used to predict the effect for the acute oral data sets not used in this calibration. The prediction results for deltamethrin are shown in Figure 5. The predictions of the shown BufferGUTS-CA-SD model have an NRMSE of 27.6% (20.7%–35.0%), and the overall shape of the prediction follows the unseen data points well. However, the effect of higher exposure concentrations on mortality is underestimated. For ethiprole (BufferGUTS-DA-SD, Figure S13), the NRMSE of 26.0% (22.5%–29.7%) is comparable to deltamethrin, but the model tends to overpredict effects, especially for intermediate concentrations. The model also predicts a high mortality for these intermediate concentrations beyond the observation period of acute oral tests. Although there are no data points in this period to disprove this prediction, it appears to highly overpredict mortality after the first days. The other three substances, imidacloprid (BufferGUTS-CA-IT, Figure S14), tetraniliprole (BufferGUTS-CA-SD, Figure S15), and thiacloprid (BufferGUTS-CA-SD, Figure S16), all show a reasonable agreement of model predictions with unseen acute oral data points. For imidacloprid with an NRMSE of 12.0% (11.1%–12.9%) and tetraniliprole with an NRMSE of 10.7% (9.9%–11.7%), the predictions over time also show no tendency to overpredict or underpredict effects. Thiacloprid, while having a comparable NRMSE of 12.5% (10.4%–14.9%), overpredicts the effects on mortality for intermediate concentrations, similar to ethiprole. For both of these substances, the chronic oral data sets used for calibration mainly contain exposure concentrations with no effect on survival and only two concentrations with mortality greater than 20%. Furthermore, the highest exposure concentration in these data sets leads to almost linear mortality with about 100% mortality after 10 days. Such data alone thus appear to be insufficient to identify the survival curve for predictions. The chronic oral data set for tetraniliprole also only has two concentration levels with a mortality above 20%, but the highest concentration leads to full mortality after only four to 5 days. This results in a well-shaped survival curve and thus enables the selected model (BufferGUTS-CA-SD) to better predict the unseen data.

**Figure 5:**
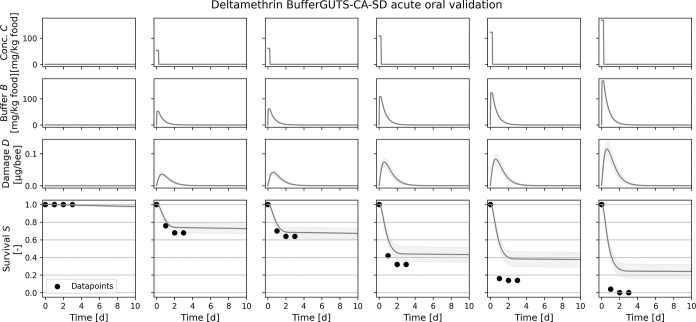
Prediction of unseen data for deltamethrin using the BufferGUTS-CA-SD model with concentration addition (CA) and stochastic death (SD). The used model was calibrated to just acute contact and chronic oral exposure data and then used to predict the unseen acute oral data.

### Validation Scenario Definition

In the latest scientific opinion of TKTD models by the European Food Safety Authority (EFSA), a certain calibration and validation procedure for the use of GUTS as a refinement method in tier 2 of the aquatic risk assessment is required.^12^ The idea is to calibrate the model on constant exposure and then validate it using data from survival observations under multiple exposure pulses because this is the intended use of calibrated GUTS models. This procedure is already part of aquatic GUTS applications^7,9^ and is required to test the performance of the model for unseen exposure scenarios. For terrestrial applications, the procedure might need adjustments. The topical exposure used in contact experiments, for example, poses challenges from an experimental standpoint. Topical applications cannot be performed in a “chronic” way, and multiple contact exposure peaks would require additional sedation of the individuals. Such additional applications introduce further stress beyond the substance effect, hence, reducing the comparability of experiments with other exposure scenarios and the control. Therefore, validation experiments should include only topical applications at the beginning of the experiments, where individuals have to be sedated anyway to sort them into their experimental containers. However, experiments analyzing the effect of multiple topical applications are interesting to verify the model assumptions based on single-exposure experiments.^29,30^ For oral exposures, the exposure can be designed as desired because the food solution in the containers can be changed with less effort. Based on these considerations and the data sets used in the calibration (acute contact, acute, and chronic oral), we suggest that suitable validation data sets should combine contact exposures with oral exposures of exposure durations that were not used for calibration. Therefore, we propose oral exposures of 1 and 2 days for our validation scenario predictions. Figure 6 shows the survival predictions for different combinations of contact and oral exposures for the same individuals for deltamethrin. Similar predictions for the other substances are shown in Figures S17–S21. With such validation data sets, existing models combining multiple uptake routes could be truly validated for the prediction of nonstandard, combined exposure scenarios.

**Figure 6:**
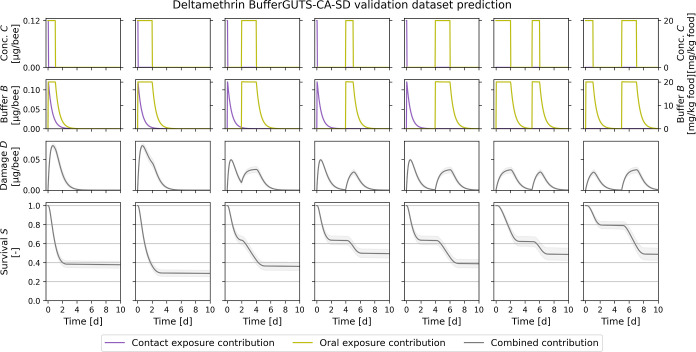
Predictions for theoretical validation experiments for deltamethrin combining contact exposure (0.12 μg a.i./bee) and oral (20 mg a.i./kg food) exposures over 1 or 2 days and different times between exposure peaks for the BufferGUTS-CA-SD model with concentration addition (CA) and stochastic death (SD).

### Prospects for the Application in Risk Assessment

Our newly developed BufferGUTS model offers several advantages over the existing BeeGUTS model,^13^ particularly in generating more realistic risk assessments for above-ground nontarget arthropods (NTAs). While BeeGUTS assumes that all exposure routes contribute equally to toxicity, BufferGUTS introduces the use of calibrated weights to reflect the potentially different contributions of each route. This addition enables explicit analysis and quantification of the importance of individual uptake routes, enabling a more focused risk assessment.

Moreover, the BeeGUTS standard parameterization assumes the same absorption through the exoskeleton and identical uptake behavior within the gut for all compounds. In contrast, BufferGUTS allows these parameters to be calibrated on a substance-by-substance basis, informed directly by experimental data. This flexibility means that BufferGUTS can be reliably adapted for substances where the simplifying assumptions of BeeGUTS lead to suboptimal calibrations^6^ or unsuccessful calibrations. This was, for example, the case for two of the substance data sets used in this study and the BeeGUTS study.^13^ The BeeGUTS models were unable to provide calibration results across all uptake types for imidacloprid and tetraniliprole data sets due to inconsistencies in LD50/LC50 values across exposure routes (see BeeGUTS Supporting Information^13^). In our study, we found that assuming similar effect kinetics across uptake routes was justified for four out of the five substances examined, while assuming the same contributions to the effects was valid for only two substances.

Furthermore, BeeGUTS interpolation and effect estimation are only predefined for standard exposure types (acute oral, acute contact, chronic oral), so that for the applicability to nonstandard scenarios or combined exposures (e.g., prolonged oral exposure over 5 days), case-by-case adjustments are required. BufferGUTS, on the contrary, can incorporate arbitrary exposure durations and combinations of exposure routes, significantly expanding its utility in realistic risk assessment contexts.

Based on these differences, we recommend BufferGUTS as the preferred approach for the risk assessment of above-ground NTAs when data from one or more exposure routes are available. The appropriate species and substance model should then be selected by the model choice procedure outlined in the Materials and Methods section. This can be done for substances and above-ground invertebrate species without further adaptation of the model, thus enabling the inclusion of new species into the risk assessment with no species-specific information available. For species where validated species-specific BeeGUTS parameters exist (^13^ and selected non-Apis bees^14^), BeeGUTS can be used for extrapolation from one standard test to another.

Independent of the exact model, TKTD models share the fundamental ability to incorporate multiple data sets into one calibration, thus enabling the digestion of all available data for a species and substance into one set of model parameters. Analyzing the BufferGUTS model parameters additionally offers insight into the varying effect speeds and contributions of different uptake routes, enabling a deeper understanding of their impacts. Having model representations of species and substance combinations enables a more robust derivation of toxicity end points, such as LC50 values, for any time point and the prediction of effects for nonstandard exposure profiles.^12^ To further deepen this understanding, future research on TKTD model applications on NTAs should broaden its scope by testing additional species and substances with different modes of action as well as a range of uptake routes and their combinations. Such analyses and data sets will further evaluate and refine the robustness and generality of TKTD modeling approaches for NTAs. Overall, our findings support the integration of toxicokinetic–toxicodynamic (TKTD) models such as BufferGUTS into environmental risk assessment workflows to enhance both the accuracy and ecological relevance of results and thus better safeguard terrestrial nontarget arthropod populations.

## Supplementary Material



## Data Availability

Python code used to calibrate the models: https://gitlab.uni-osnabrueck.de/fschunck/bee_study. This code was just developed and used for this study and is no longer maintained or developed further. Python package to calibrate BufferGUTS to own data: https://gitlab.uni-osnabrueck.de/memuos/mempyguts. This package is maintained further and should be used for new calibrations. The full honeybee test reports listed in Table 1 can be requested by sending an email with the report number to cropscience-transparency@bayer.com.

## Notes

Model equations, definitions of all parameters and state variables, calibrated parameter values, and figures for all substances showing the calibration results, prediction of unseen data and validation scenario suggestions (PDF)
