# Supplementary material for: Combined Modeling of Multiple Exposure Routes for Terrestrial Arthropods Using the Toxicokinetic–Toxicodynamic BufferGUTS Model
Source: Environ Sci Technol. 2025 Jul 29;59(31):16314–23. doi: 10.1021/acs.est.5c03925 (PMC12355943; doi:10.1021/acs.est.5c03925)
Supplement: Supplementary file 1 [file es5c03925_si_001.pdf]

Supporting Information for: Combined Modeling of Multiple Exposure Routes for Terrestrial Arthropods using the Toxicokinetic-Toxicodynamic BufferGUTS Model

Leonhard Urs Bürger[1,*], Florian Schunck[1], Andreas Focks[1]

[1] Osnabrück University, Barbarastr. 12, 49076 Osnabrück, Germany

[*] Corresponding author, leonhard.buerger@uni-osnabrueck.de

This document contains 21 Figures and 2 Tables on 19 pages.

Contents

1 Model variant equations **S3**

1.1 Exposure route combinations S3

1.2 Death mechanisms S3

2 Additional figures **S5**

2.1 Calibrated parameter values S5

2.2 Calibration results for all substances S7

2.3 Prediction of unseen data S13

2.4 Validation scenario prediction S15

List of Figures

Figure S1 Calibrated Parameters for the deltamethrin dataset. S5
Figure S2 Calibrated Parameters for the ethiprole dataset. S5
Figure S3 Calibrated Parameters for the imidacloprid dataset. S6
Figure S4 Calibrated Parameters for the tetraniliprole dataset. S6
Figure S5 Calibrated Parameters for the thiacloprid dataset. S6
Figure S6 All calibration results for the deltamethrin dataset. S7
Figure S7 All calibration results for the ethiprole dataset. S8
Figure S8 Calibration results parts 1 to 3 for the imidacloprid dataset. S9
Figure S9 Calibration results parts 4 and 5 for the imidacloprid dataset. S10
Figure S10 All calibration results for the tetraniliprole dataset. S11
Figure S11 All calibration results for the thiacloprid dataset. S12
Figure S12 Prediction of unseen data for deltamethrin using the BufferGUTS-CA-SD model. S13
Figure S13 Prediction of unseen data for ethiprole using the BufferGUTS-DA-SD model. . S13
Figure S14 Prediction of unseen data for Imidaclorpid using the BufferGUTS-CA-IT model. S14
Figure S15 Prediction of unseen data for tetraniliprole using the BufferGUTS-CA-SD model. S14
Figure S16 Prediction of unseen data for thiacloprid using the BufferGUTS-CA-SD model. S15
Figure S17 Predictions for theoretical validation experiments for deltamethrin with the BufferGUTS-CA-SD model. S15
Figure S18 Predictions for theoretical validation experiments for ethiprole with the BufferGUTS-DA-SD model. S16
Figure S19 Predictions for theoretical validation experiments for imidacloprid with the BufferGUTS-CA-IT model. S16
Figure S20 Predictions for theoretical validation experiments for tetraniliprole with the BufferGUTS-CA-SD model. S17
Figure S21 Predictions for theoretical validation experiments for thiacloprid with the BufferGUTS-CA-SD model. S17

List of Tables

Table S1 Table of all state variables and parameters used the models and its units. S4
Table S2 Table of all calibrated parameters for all models and substances. S18

1 Model variant equations

All models use the same same set of parameters described in Table S1.

1.1 Exposure route combinations

Concentration addition (CA) models:

$$\frac{dB_{1\ldots N}(t)}{dt} = k\left(C_{1\ldots N}(t) - B_{1\ldots N}(t)\right) \text{ with } k = \begin{cases} \eta \text{ for } B_{1\ldots N}(t) \leq C_{1\ldots N}(t), \\ k_d \text{ for } B_{1\ldots N}(t) > C_{1\ldots N}(t) \end{cases} \quad \text{(Eq. S1)}$$

$$\frac{dB_{\sum}(t)}{dt} = \sum_{i=1}^{N} \frac{dB_i(t)}{dt} \quad \text{(Eq. S2)}$$

$$\frac{dD(t)}{dt} = k_d(B_{\sum} - D(t)) \quad \text{(Eq. S3)}$$

Damage addition (DA) models:

$$\frac{dB_{1\ldots N}(t)}{dt} = k\left(C_{1\ldots N}(t) - B_{1\ldots N}(t)\right) \text{ with } k = \begin{cases} \eta \text{ for } B_{1\ldots N}(t) \leq C_{1\ldots N}(t), \\ k_{d1\ldots N} \text{ for } B_{1\ldots N}(t) > C_{1\ldots N}(t) \end{cases} \quad \text{(Eq. S4)}$$

$$\frac{dD_{1\ldots N}(t)}{dt} = k_{d1\ldots N}(B_{1\ldots N}(t) - D_{1\ldots N}(t)) \quad \text{(Eq. S5)}$$

$$\frac{dD_{\sum}(t)}{dt} = \sum_{i=1}^{N} \frac{dD_i(t)}{dt} \quad \text{(Eq. S6)}$$

1.2 Death mechanisms

Used variable damage D can be either the damage D of CA models or the damage sum $D_{\sum}$ of DA models. The death mechanism are equal to Jager and Ashauer (2017, pp. 50-51) [2].

Stochastic death (SD) models:

$$h_z = b \cdot \max(0, D(t) - z) + h_b \quad \text{(Eq. S7)}$$

$$\frac{dS(t)}{dt} = -h_z S(t) \quad \text{(Eq. S8)}$$

Individual tolerance (IT) models:

With f as a log-logistic distribution of the individual threshold with median α and shape parameter β and F the cumulative distribution of the same function.

$$D_{max} = \max_{0 \leq \tau \leq t} D(\tau) \quad \text{(Eq. S9)}$$

$$S_b = \exp(-h_b \cdot t) \quad \text{(Eq. S10)}$$

$$S = S_b \int_{D_m}^{\infty} f(z, \alpha, \beta) dz = S_b(1 - F(D_{max})) \quad \text{(Eq. S11)}$$

Table S1. Table of all state variables and parameters used the models and its units. Unit in reduced GUTS models often relate to the exposure unit, thus $unit_n$ corresponds to the unit of exposure route n.

Variable	Description	Unit
$C_{1...N}$	Discretized exposure via one of N exposure routes	$unit_n$
$B_{1...N}$	Buffer state between the external concentration C and the internal damage D. The buffer can be interpreted as e.g. substance on the exoskeleton or in the stomach of an individual.	$unit_n$
$B_{\sum}$	Buffer sum combining all buffers $B_{1...N}$ using weights $w_{1...N}$ into the main unit of exposure route 1. Only used in concentration addition (CA) models.	$unit_1$
D	Damage of the stressor and the state to feed into the death mechanisms. Only for concentration addition (CA) models	$unit_1$
$D_{1...N}$	Damage from each uptake route n individually. Only for damage addition (DA) models.	$unit_n$
$D_{\sum}$	Damage sum combining all damages $D_{1...N}$ using weights $w_{1...N}$ into the main unit of exposure route 1. Only used in damage addition (DA) models.	$unit_1$
S	Survival rate over time resulting from the death mechanism.	-
η	Buffer speed constant that governs the speed with which the buffer is filled. We used $eta = 24000$, but other eta values $\gg 1$ lead to comparable results [1].	$1/d$
$w_{1...N}$	Weights to covert all exposures into the unit of exposure route 1, thus weight w_1 of exposure route 1 is one.	$unit_1/unit_n$
k_d	Dominant rate constant governing the depletion of the buffer B and uptake of damage D. For damage addition (DA) models each uptake route n has its own rate k_{dn}.	$1/d$
z	Threshold of stochastic death (SD) models. Only damage D amounts above the threshold result in mortality.	$unit_1$
b	Killing rate of stochastic death (SD) models. All damage D above the threshold z results in mortality according to the killing rate.	$1/d/unit_1$
α	Median of the log-logistic threshold distribution function of individual threshold (IT) models. If the individual threshold is exceeded, the individual dies.	$unit_1$
β	Shape parameter of the log-logistic threshold distribution function of individual threshold (IT) models. Higher β values result in a wider spread of individual thresholds and smaller values in a narrow distribution of thresholds.	-
h_b	Background mortality independent of any stressor. It is modeled as a first order decline.	$1/d$

2 Additional figures

2.1 Calibrated parameter values

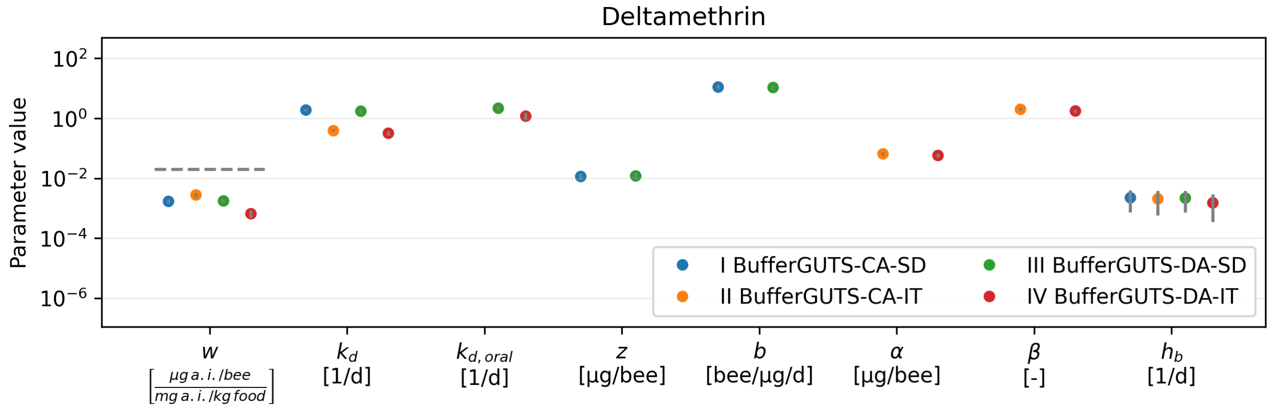

Figure S1. Calibrated Parameters for all BufferGUTS model variants for the deltamethrin dataset. Uncertainties shown are the 95% credibility intervals and the dashed horizontal line for the weights w corresponds to the 20 mg food/bee used to convert acute oral doses to food concentrations in the data pre-processing.

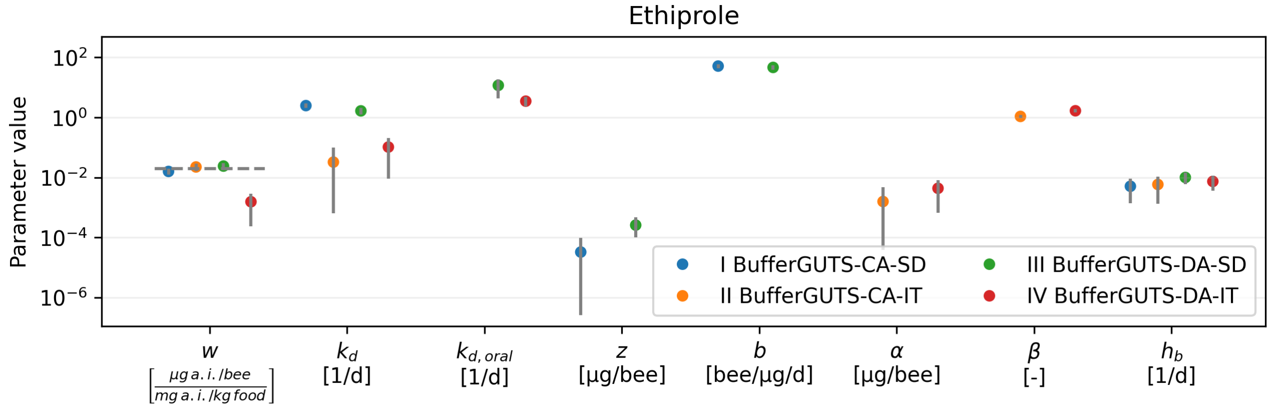

Figure S2. Calibrated Parameters for all BufferGUTS model variants for the ethiprole dataset. Uncertainties shown are the 95% credibility intervals and the dashed horizontal line for the weights w corresponds to the 20 mg food/bee used to convert acute oral doses to food concentrations in the data pre-processing.

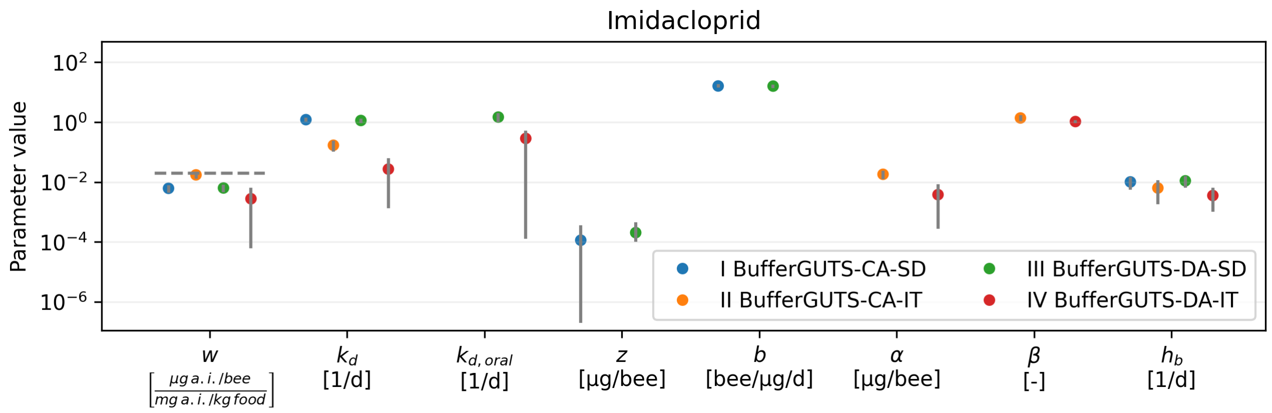

Figure S3. Calibrated Parameters for all BufferGUTS model variants for the imidacloprid dataset. Uncertainties shown are the 95% credibility intervals and the dashed horizontal line for the weights w corresponds to the 20 mg food/bee used to convert acute oral doses to food concentrations in the data pre-processing.

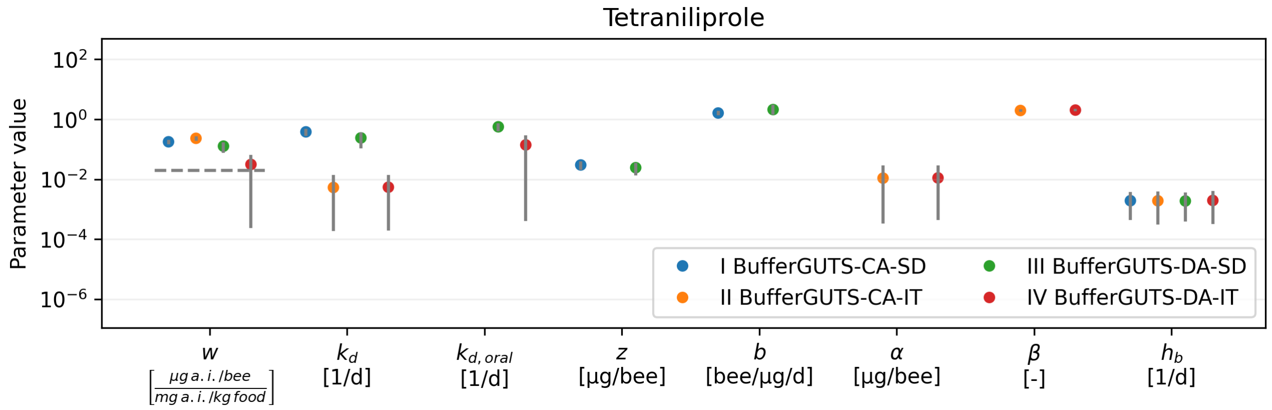

Figure S4. Calibrated Parameters for all BufferGUTS model variants for the tetraniliprole dataset. Uncertainties shown are the 95% credibility intervals and the dashed horizontal line for the weights w corresponds to the 20 mg food/bee used to convert acute oral doses to food concentrations in the data pre-processing.

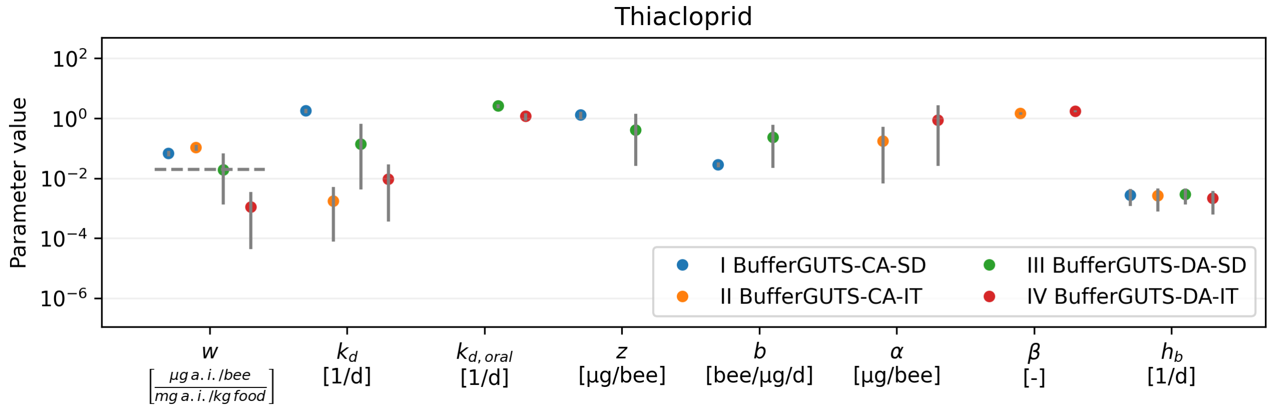

Figure S5. Calibrated Parameters for all BufferGUTS model variants for the thiacloprid dataset. Uncertainties shown are the 95% credibility intervals and the dashed horizontal line for the weights w corresponds to the 20 mg food/bee used to convert acute oral doses to food concentrations in the data pre-processing.

2.2 Calibration results for all substances

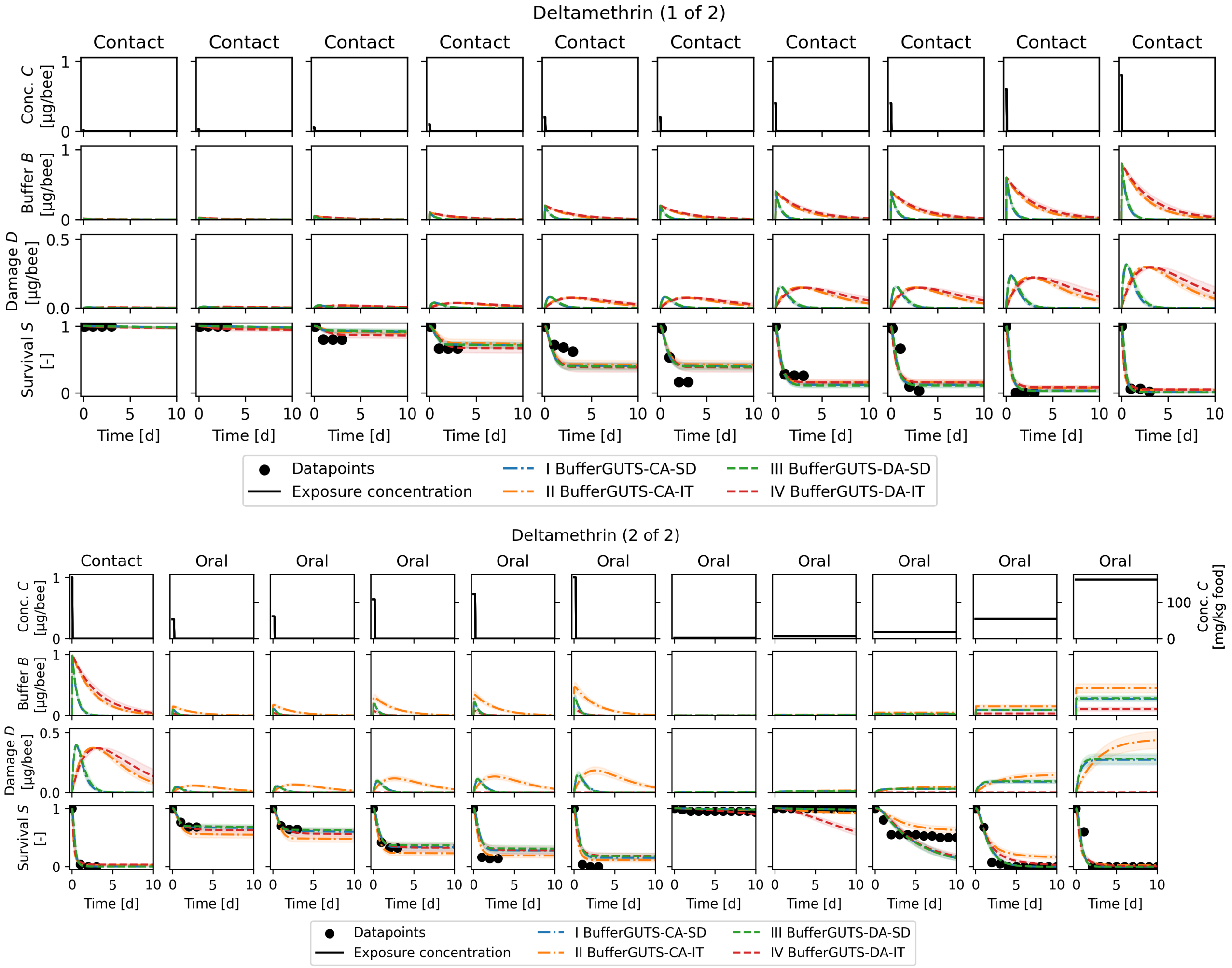

Figure S6. All calibration results for deltamethrin for the four BufferGUTS variants with concentration addition (CA) or damage addition (DA) exposure route combination and stochastic death (SD) or individual tolerance (IT) death mechanisms. Uncertainties shown are the 95% credibility intervals. Exposure route weights w are already applied when concentrations C are converted into the buffer B in this figure to ease readability.

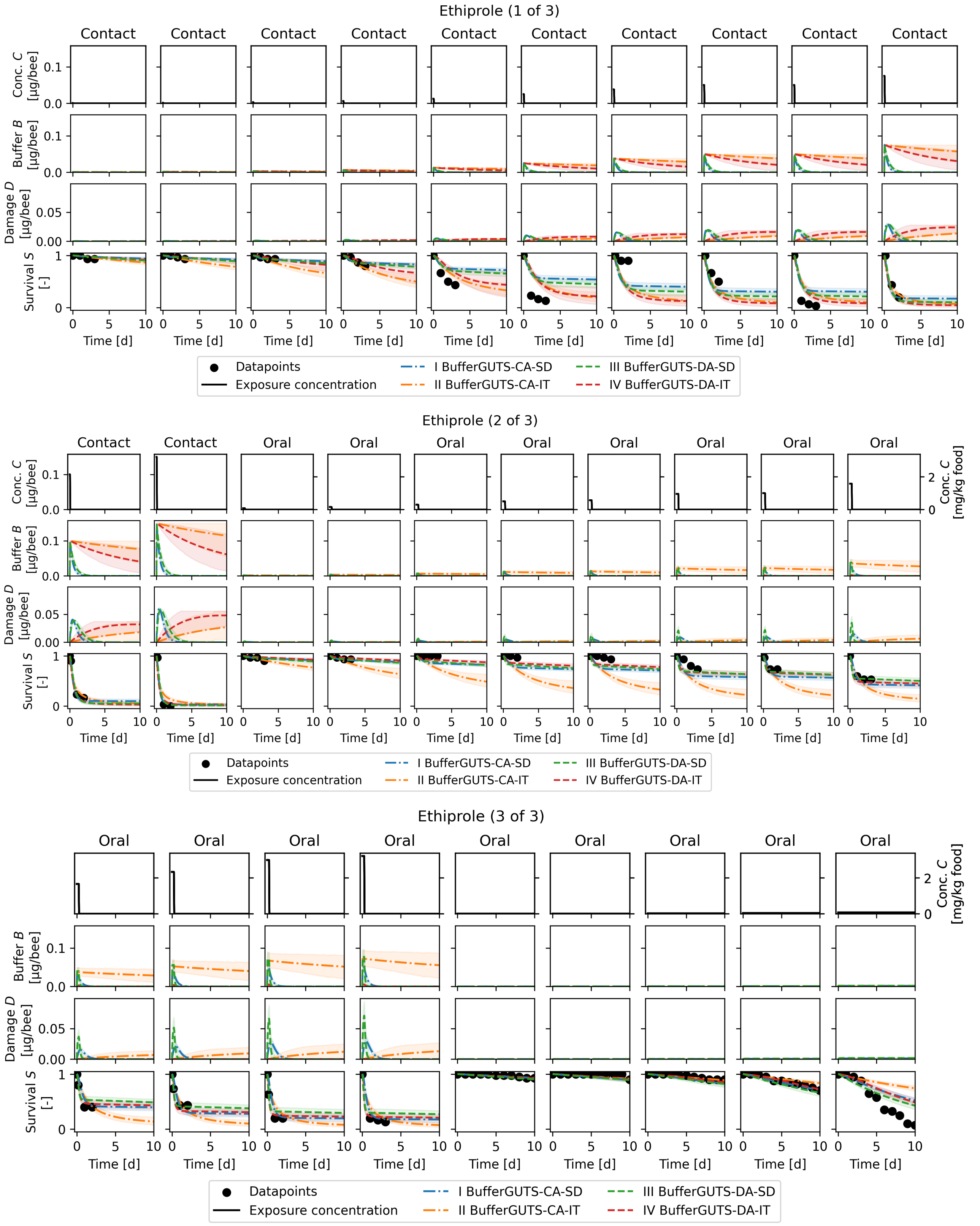

Figure S7. All calibration results for ethiprole for the four BufferGUTS variants with concentration addition (CA) or damage addition (DA) exposure route combination and stochastic death (SD) or individual tolerance (IT) death mechanisms. Uncertainties shown are the 95% credibility intervals. Exposure route weights w are already applied when concentrations C are converted into the buffer B in this figure to ease readability.

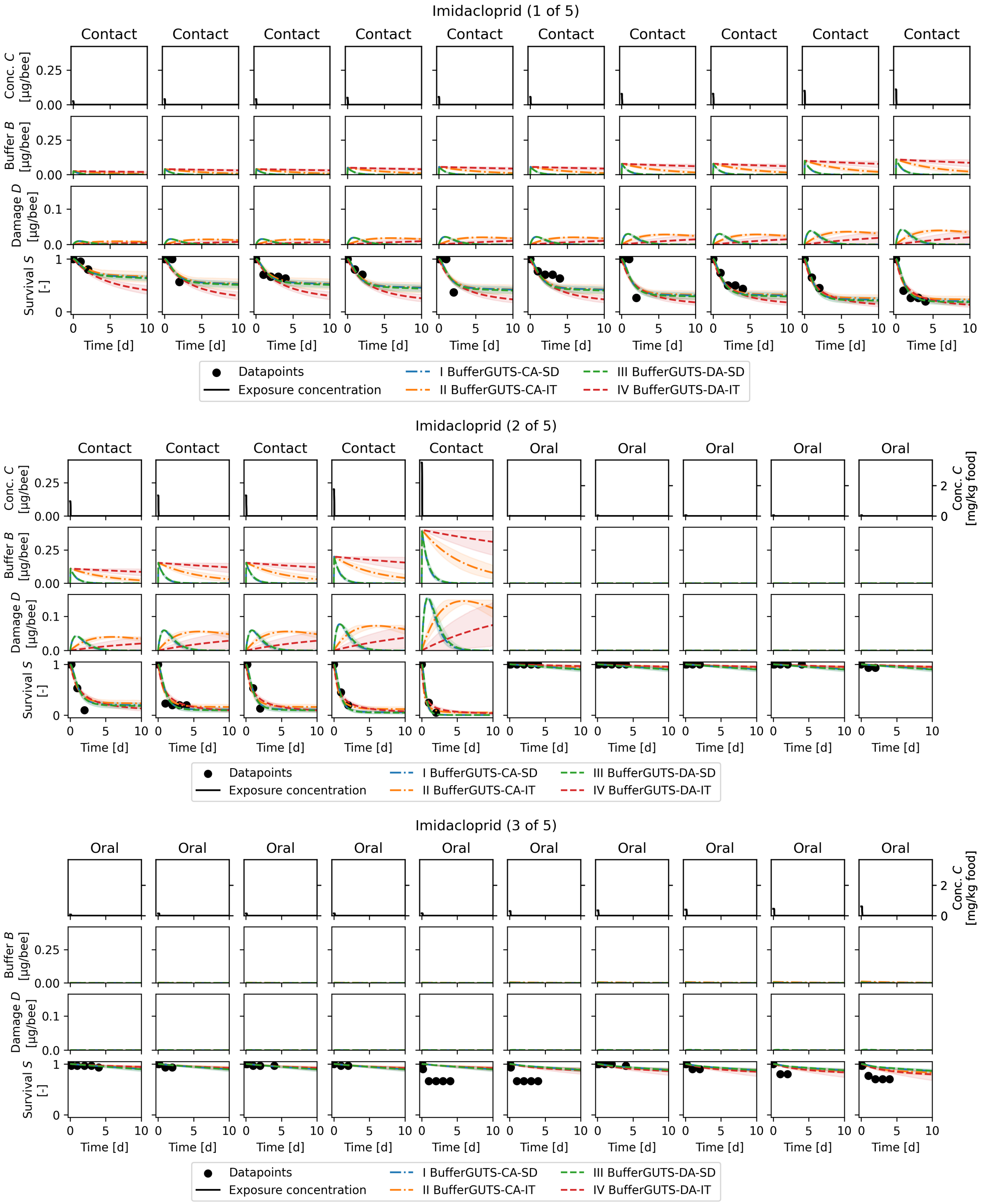

Figure S8. Calibration results parts 1 to 3 for imidacloprid for the four BufferGUTS variants with concentration addition (CA) or damage addition (DA) exposure route combination and stochastic death (SD) or individual tolerance (IT) death mechanisms. Uncertainties shown are the 95% credibility intervals. Exposure route weights w are already applied when concentrations C are converted into the buffer B in this figure to ease readability.

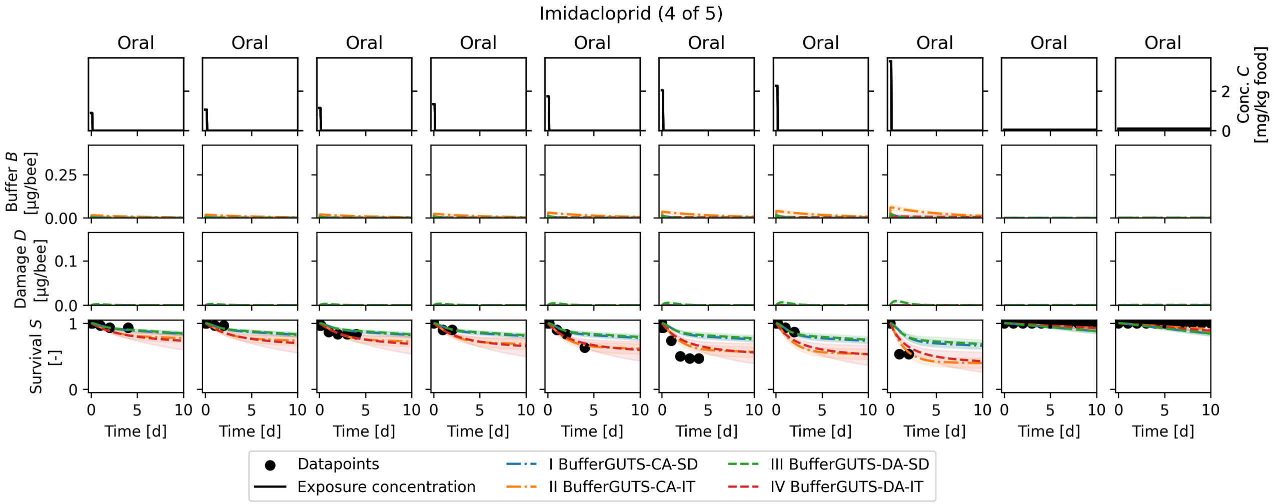

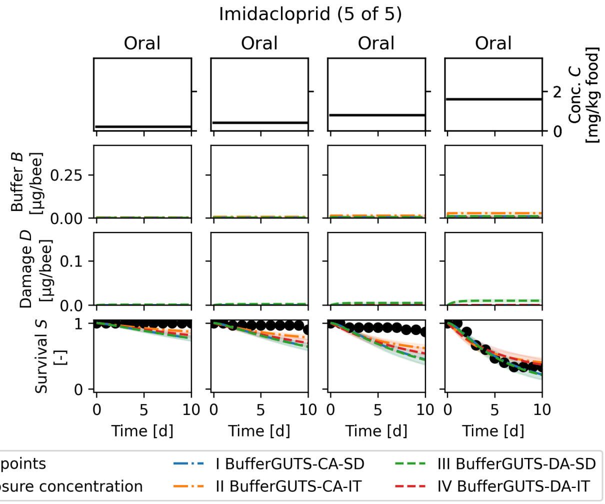

Figure S9. Calibration results parts 4 and 5 for imidacloprid for the four BufferGUTS variants with concentration addition (CA) or damage addition (DA) exposure route combination and stochastic death (SD) or individual tolerance (IT) death mechanisms. Uncertainties shown are the 95% credibility intervals. Exposure route weights w are already applied when concentrations C are converted into the buffer B in this figure to ease readability.

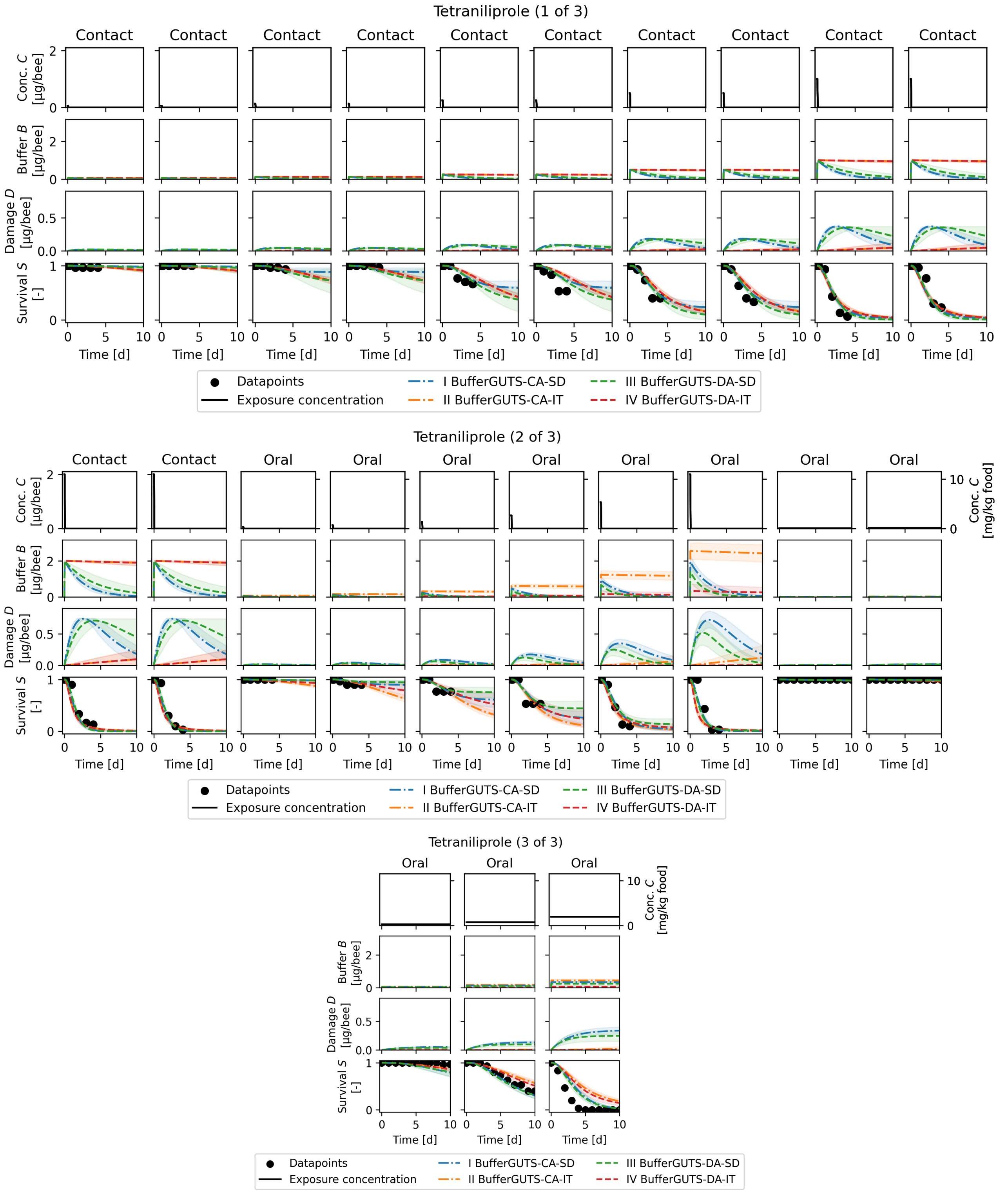

Figure S10. All calibration results for tetraniliprole for the four BufferGUTS variants with concentration addition (CA) or damage addition (DA) exposure route combination and stochastic death (SD) or individual tolerance (IT) death mechanisms. Uncertainties shown are the 95% credibility intervals. Exposure route weights w are already applied when concentrations C are converted into the buffer B in this figure to ease readability.

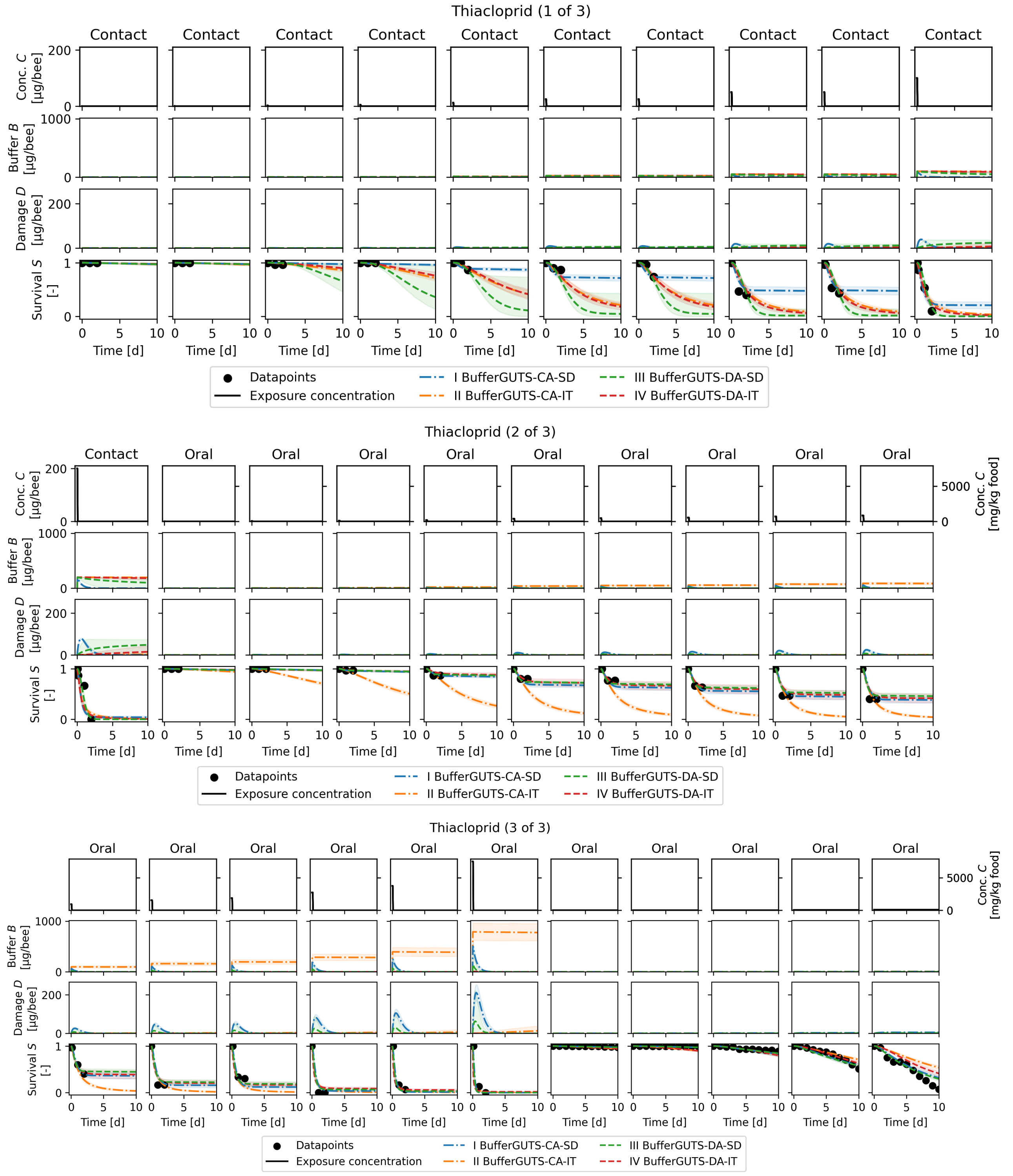

Figure S11. All calibration results for thiacloprid for the four BufferGUTS variants with concentration addition (CA) or damage addition (DA) exposure route combination and stochastic death (SD) or individual tolerance (IT) death mechanisms. Uncertainties shown are the 95% credibility intervals. Exposure route weights w are already applied when concentrations C are converted into the buffer B in this figure to ease readability.

2.3 Prediction of unseen data

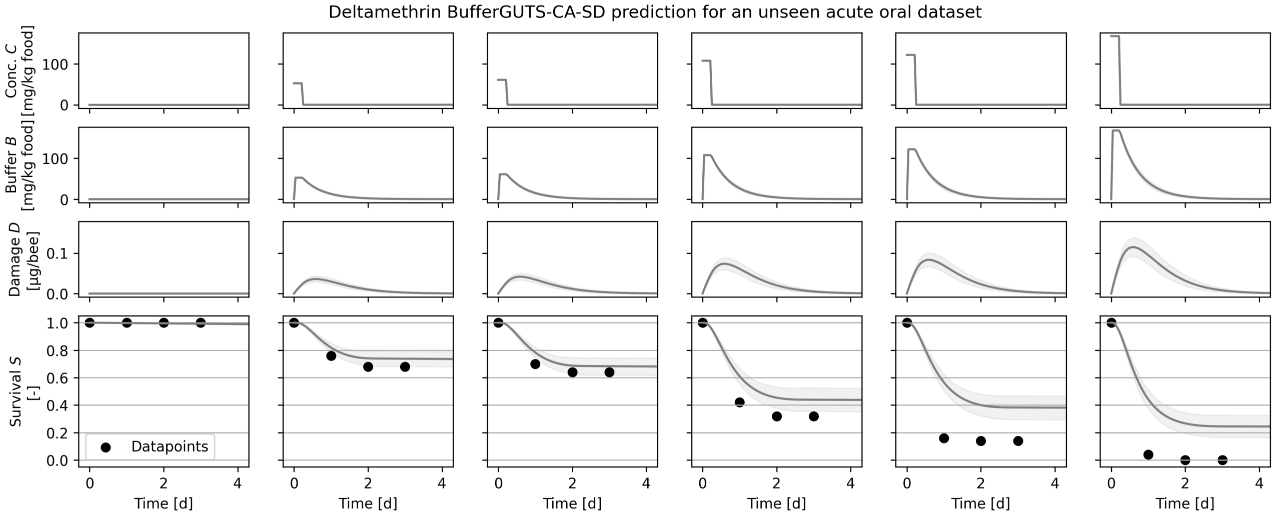

Figure S12. Prediction of unseen data for deltamethrin using the BufferGUTS-CA-SD model with concentration addition (CA) and stochastic death (SD). Used model was calibrated to just acute contact and chronic oral exposure data and then used to predict the unseen acute oral data. This figure is also included in the main manuscript but repeated here for better comparison with the other prediction results.

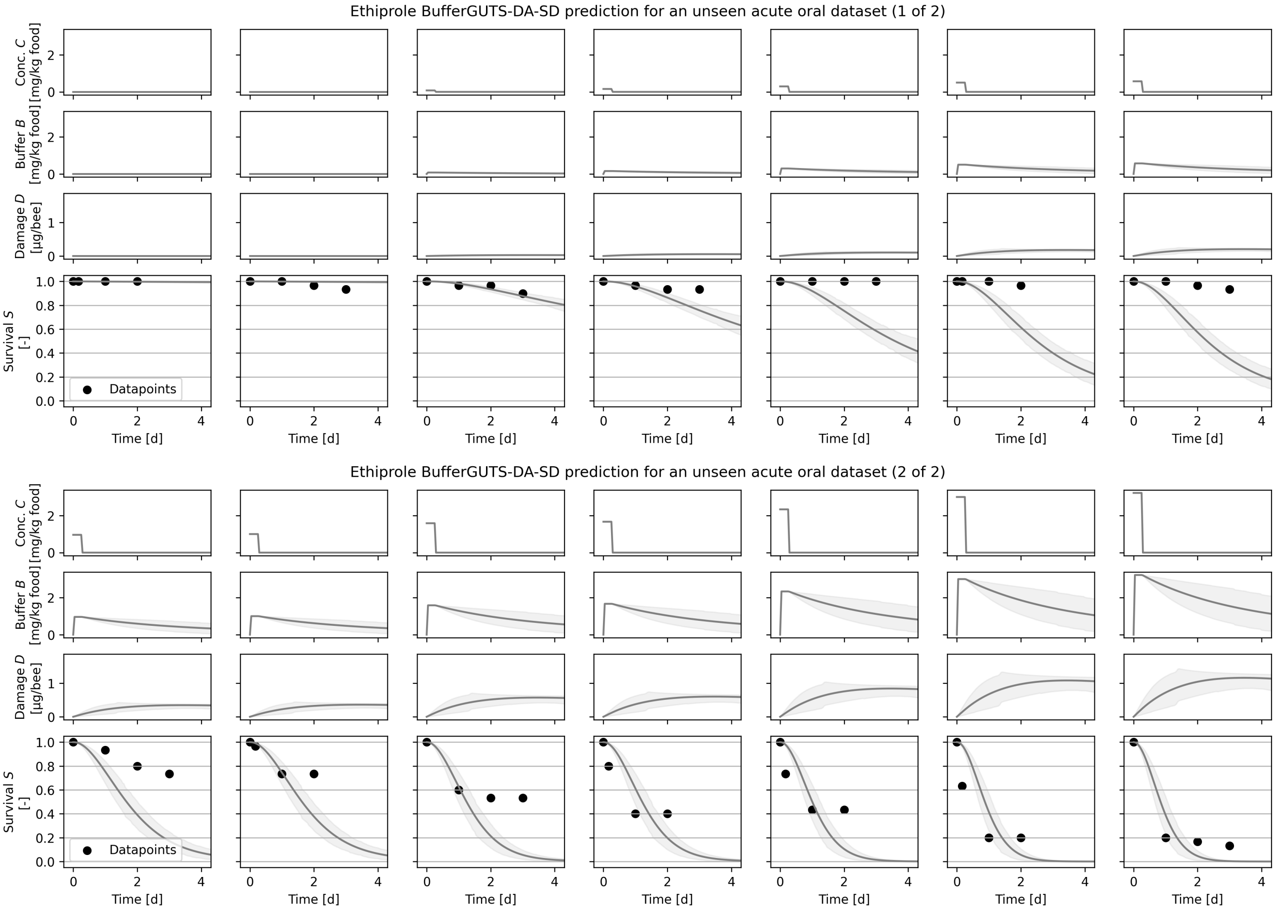

Figure S13. Prediction of unseen data for ethiprole using the BufferGUTS-DA-SD model with damage addition (DA) and stochastic death (SD). Used model was calibrated to just acute contact and chronic oral exposure data and then used to predict the unseen acute oral data.

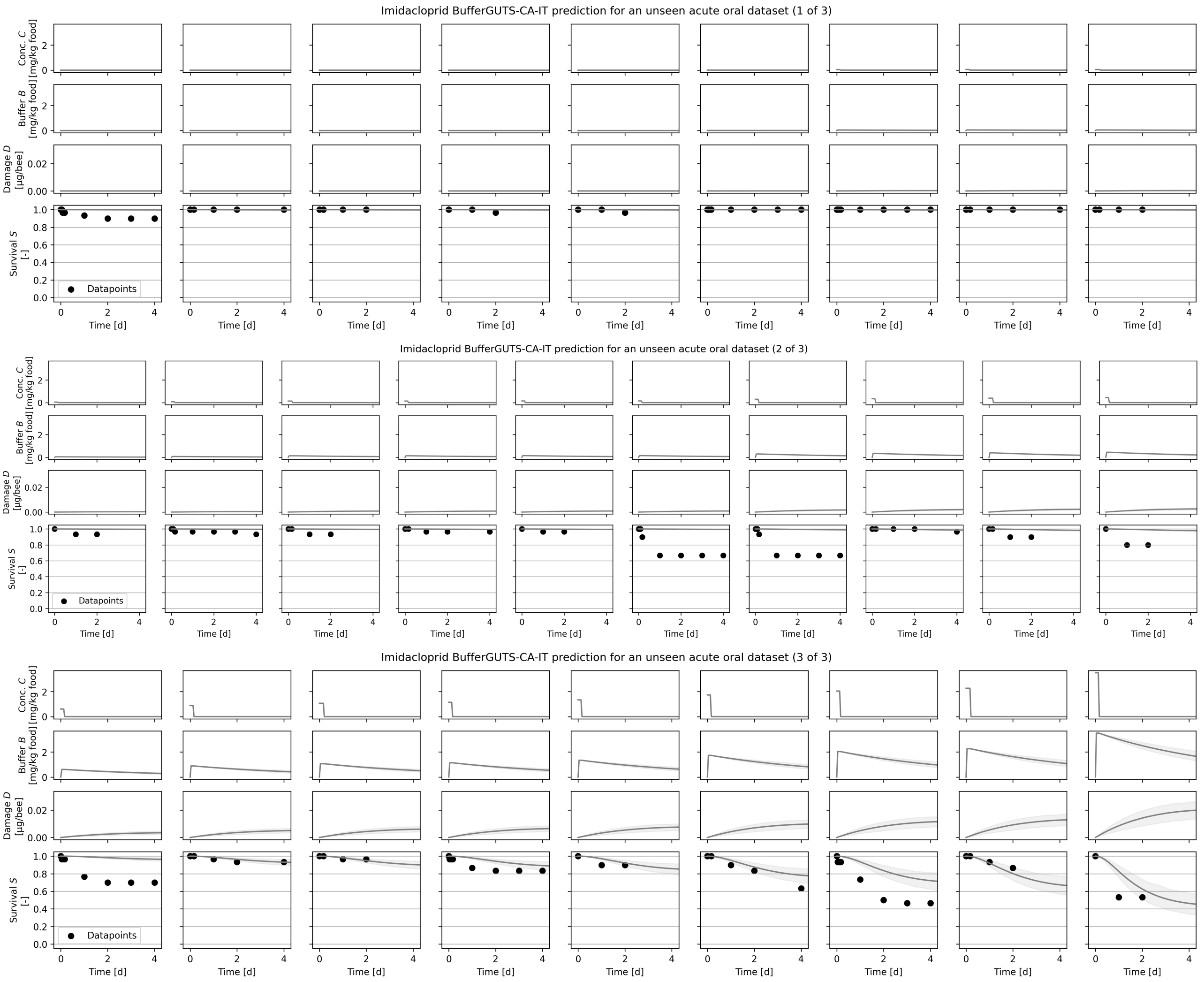

Figure S14. Prediction of unseen data for imidacloprid using the BufferGUTS-CA-IT model with concentration addition (CA) and individual tolerance (IT). Used model was calibrated to just acute contact and chronic oral exposure data and then used to predict the unseen acute oral data.

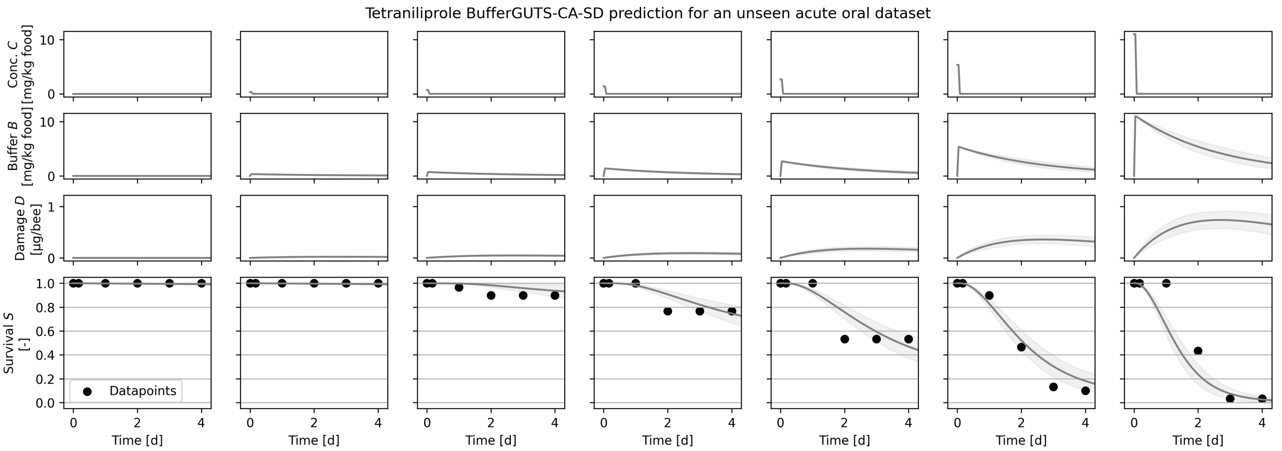

Figure S15. Prediction of unseen data for tetraniliprole using the BufferGUTS-CA-SD model with concentration addition (CA) and stochastic death (SD). Used model was calibrated to just acute contact and chronic oral exposure data and then used to predict the unseen acute oral data.

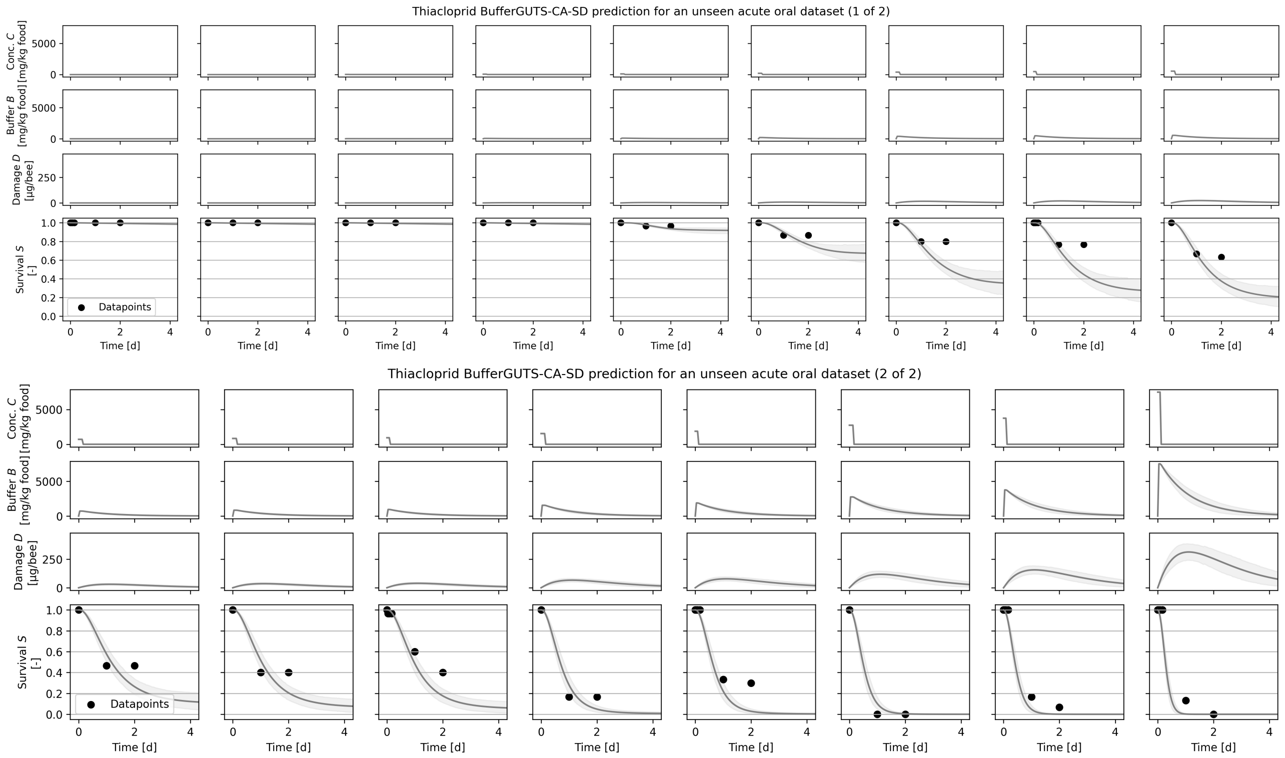

Figure S16. Prediction of unseen data for thiacloprid using the BufferGUTS-CA-SD model with concentration addition (CA) and stochastic death (SD). Used model was calibrated to just acute contact and chronic oral exposure data and then used to predict the unseen acute oral data.

2.4 Validation scenario prediction

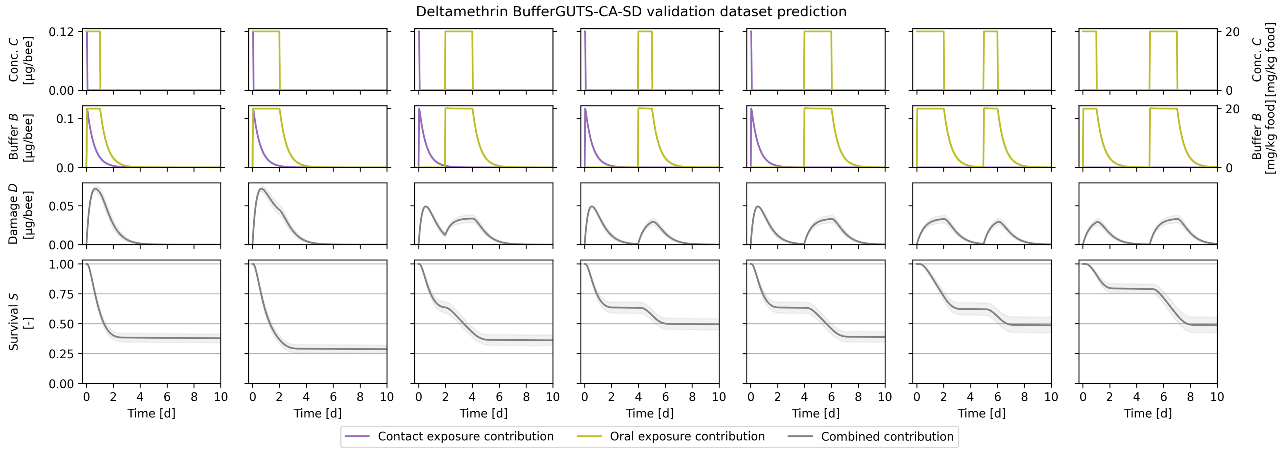

Figure S17. Predictions for theoretical validation experiments for deltamethrin combining contact exposure (0.12 μg a.i./bee) and oral (20 mg a.i./kg food) exposures over one or two days and different times between exposure peaks for the BufferGUTS-CA-SD model with concentration addition (CA) and stochastic death (SD). This figure is also included in the main manuscript but repeated here for better comparison with the other prediction results.

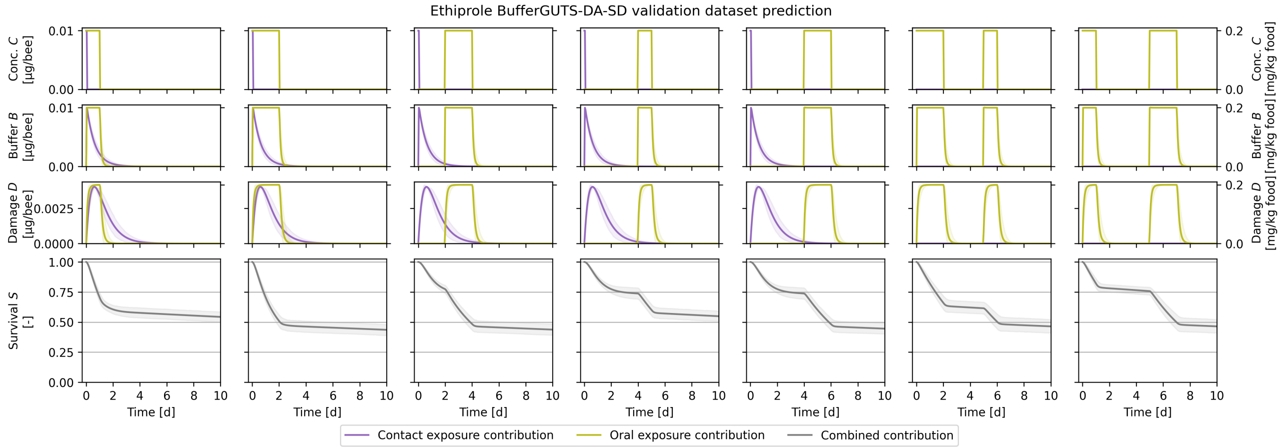

Figure S18. Predictions for theoretical validation experiments for ethiprole combining contact exposure (0.01 μg a.i./bee) and oral (0.2 mg a.i./kg food) exposures over one or two days and different times between exposure peaks for the BufferGUTS-CA-SD model with damage addition (DA) and stochastic death (SD).

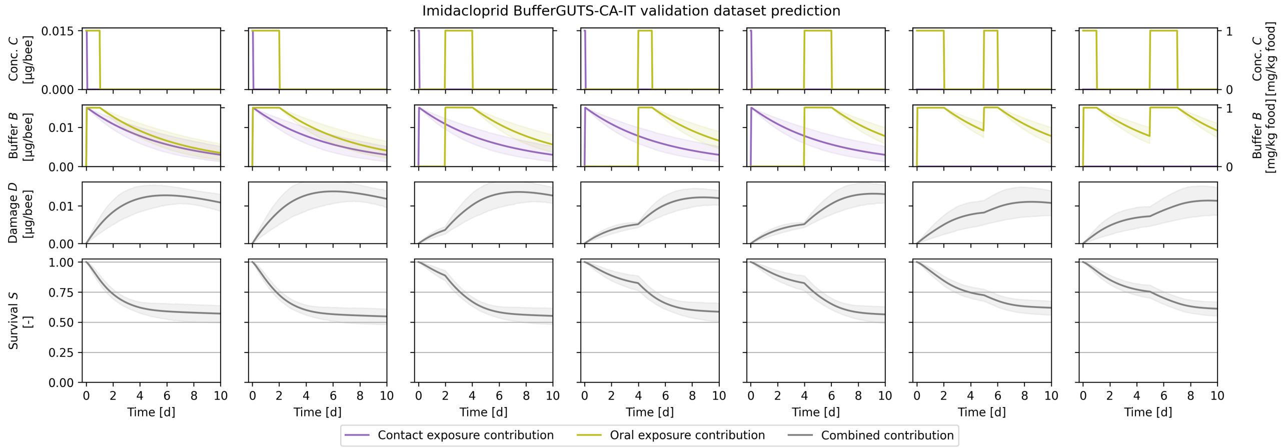

Figure S19. Predictions for theoretical validation experiments for imidacloprid combining contact exposure (0.015 μg a.i./bee) and oral (1.0 mg a.i./kg food) exposures over one or two days and different times between exposure peaks for the BufferGUTS-CA-SD model with concentration addition (CA) and individual tolerance (IT).

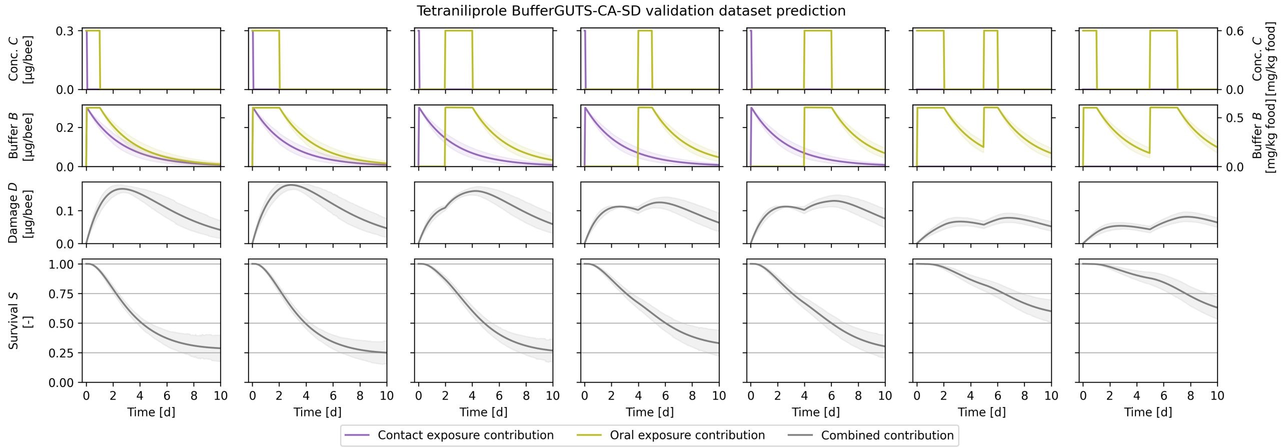

Figure S20. Predictions for theoretical validation experiments for tetraniliprole combining contact exposure (0.3 μg a.i./bee) and oral (0.6 mg a.i./kg food) exposures over one or two days and different times between exposure peaks for the BufferGUTS-CA-SD model with concentration addition (CA) and stochastic death (SD).

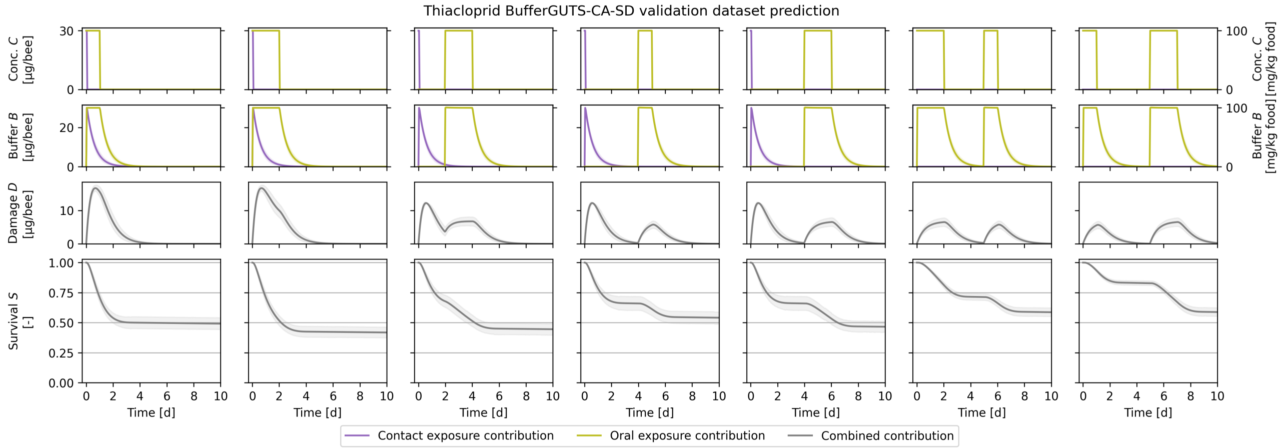

Figure S21. Predictions for theoretical validation experiments for thiacloprid combining contact exposure (30 μg a.i./bee) and oral (100 mg a.i./kg food) exposures over one or two days and different times between exposure peaks for the BufferGUTS-CA-SD model with concentration addition (CA) and stochastic death (SD).

Table S2. Table of the mean calibrated parameters for all five substances for all BufferGUTS models (CA: Concentration addition; DA: damage addition) with their death mechanisms (SD: stochastic death; IT: individual tolerance). The best model for each substance is highlighted in bold and the 95% credibility intervals of each parameter are shown in the brackets.

Substance & model	w $\left[\frac{\mu g/bee}{mg/kg\ food}\right]$	k_d [1/d]	$k_{d,oral}$ [1/d]	z [μg/bee]	b [bee/μg/d]	α [μg/bee]	β [-]	h_b [1/d]
**Deltamethrin**	**0.00169**	**1.88**	-	**0.0114**	**11**	-	-	**0.00223**
**CA-SD**	**(0.0014-0.0019)**	**(1.7-2.1)**	-	**(0.0089-0.014)**	**(9.6-12)**	-	-	**(0.00073-0.0039)**
Deltametrin	0.00276	0.386	-	-	-	0.0649	1.99	0.00206
CA-IT	(0.0023-0.0032)	(0.35-0.42)	-	-	-	(0.056-0.074)	(1.8-2.2)	(0.00057-0.0038)
Deltametrin	0.00175	1.72	2.16	0.0119	10.6	-	-	0.00217
DA-SD	(0.0015-0.002)	(1.5-1.9)	(1.9-2.5)	(0.0094-0.015)	(9.3-12)	-	-	(0.00072-0.0038)
Deltametrin	0.000653	0.317	1.18	-	-	0.0574	1.75	0.0015
DA-IT	(0.00048-0.00085)	(0.26-0.37)	(0.86-1.5)	-	-	(0.048-0.067)	(1.6-1.9)	(0.00035-0.0029)
Ethiprole	0.0158	2.48	-	3.26e-05	51.4	-	-	0.00508
CA-SD	(0.012-0.019)	(2.1-2.9)	-	(2.6e-07-9.5e-05)	(43-60)	-	-	(0.0014-0.009)
Ethiprole	0.0224	0.0325	-	-	-	0.00158	1.08	0.00584
CA-IT	(0.016-0.029)	(0.00064-0.1)	-	-	-	(3.8e-05-0.0047)	(0.95-1.2)	(0.0013-0.011)
**Ethiprole**	**0.0239**	**1.65**	**11.7**	**0.000258**	**46.4**	-	-	**0.00999**
**DA-SD**	**(0.018-0.03)**	**(1.3-2.1)**	**(4.3-18)**	**(0.0001-0.00048)**	**(39-54)**	-	-	**(0.006-0.014)**
Ethiprole	0.00155	0.103	3.48	-	-	0.00435	1.67	0.00737
DA-IT	(0.00024-0.0029)	(0.009-0.2)	(2.3-4.8)	-	-	(0.00066-0.0081)	(1.5-1.9)	(0.0036-0.012)
Imidacloprid	0.00615	1.21	-	0.000113	16.1	-	-	0.0101
CA-SD	(0.0044-0.0079)	(1-1.4)	-	(2e-07-0.00035)	(14-18)	-	-	(0.0054-0.015)
**Imidacloprid**	**0.0173**	**0.169**	-	-	-	**0.0181**	**1.38**	**0.0063**
**CA-IT**	**(0.013-0.022)**	**(0.1-0.24)**	-	-	-	**(0.012-0.024)**	**(1.1-1.7)**	**(0.0018-0.011)**
Imidacloprid	0.00631	1.14	1.47	0.000203	16	-	-	0.0109
DA-SD	(0.0045-0.0081)	(0.94-1.3)	(1-2)	(0.0001-0.00044)	(14-18)	-	-	(0.0063-0.016)
Imidacloprid	0.00275	0.027	0.285	-	-	0.00378	1.05	0.00353
DA-IT	(6e-05-0.0065)	(0.0013-0.063)	(0.00013-0.53)	-	-	(0.00028-0.0085)	(0.92-1.2)	(0.001-0.0063)
**Tetraniliprole**	**0.177**	**0.38**	-	**0.0299**	**1.62**	-	-	**0.00192**
**CA-SD**	**(0.15-0.21)**	**(0.28-0.48)**	-	**(0.021-0.039)**	**(1.3-1.9)**	-	-	**(0.00043-0.0037)**
Tetraniliprole	0.232	0.0053	-	-	-	0.0109	1.96	0.00191
CA-IT	(0.19-0.27)	(0.00019-0.014)	-	-	-	(0.00034-0.029)	(1.8-2.1)	(0.00031-0.004)
Tetraniliprole	0.127	0.239	0.561	0.0243	2.11	-	-	0.00188
DA-SD	(0.077-0.17)	(0.11-0.37)	(0.4-0.73)	(0.013-0.035)	(1.4-3)	-	-	(0.00039-0.0037)
Tetraniliprole	0.0311	0.00541	0.14	-	-	0.0111	2.03	0.00196
DA-IT	(0.00024-0.065)	(0.00019-0.014)	(0.0004-0.29)	-	-	(0.00044-0.029)	(1.8-2.2)	(0.00032-0.004)
**Thiacloprid**	**0.067**	**1.77**	-	**1.29**	**0.028**	-	-	**0.0027**
**CA-SD**	**(0.054-0.081)**	**(1.5-2.1)**	-	**(0.91-1.7)**	**(0.023-0.033)**	-	-	**(0.0012-0.0043)**
Thiacloprid	0.105	0.00172	-	-	-	0.172	1.45	0.0026
CA-IT	(0.083-0.13)	(7.7e-05-0.0052)	-	-	-	(0.0066-0.52)	(1.3-1.6)	(0.00078-0.0046)
Thiacloprid	0.0189	0.136	2.58	0.402	0.23	-	-	0.00288
DA-SD	(0.0014-0.067)	(0.0041-0.65)	(2.2-3)	(0.026-1.4)	(0.022-0.6)	-	-	(0.0013-0.0046)
Thiacloprid	0.00109	0.00924	1.17	-	-	0.861	1.71	0.00214
DA-IT	(4.2e-05-0.0034)	(0.00035-0.029)	(0.84-1.5)	-	-	(0.026-2.7)	(1.6-1.9)	(0.00063-0.0038)

References

[1] Leonhard Urs Bürger and Andreas Focks. "From water to land—Usage of Generalized Unified Threshold models of Survival (GUTS) in an above-ground terrestrial context exemplified by honeybee survival data". In: *Environmental Toxicology and Chemistry* 44.2 (2025), pp. 589–598. ISSN: 0730-7268. DOI: 10.1093/etojnl/vgae058.

[2] Tjalling Jager and Roman Ashauer. *Modelling survival under chemical stress.* Leanpub, 2018. URL: https://leanpub.com/guts_book.
